# Taxon-specific expansion and loss of *tektins* inform metazoan ciliary diversity

**DOI:** 10.1186/s12862-019-1360-0

**Published:** 2019-01-31

**Authors:** Benjamin R. Bastin, Stephan Q. Schneider

**Affiliations:** 1 Department of Genetics, Development and Cell Biology, Iowa State University, Ames, IA USA; 2 Present Address: Institute of Cellular and Organismic Biology, Academia Sinica, No. 128, Section 2, Academia Rd, Nangang District, Taipei City, 11529 Taiwan

**Keywords:** Tektin, Ciliogenesis, Cilia, Flagella, Metazoa, Evolution, Phylogeny

## Abstract

**Background:**

Cilia and flagella are complex cellular structures thought to have first evolved in a last ciliated eukaryotic ancestor due to the conserved 9 + 2 microtubule doublet structure of the axoneme and associated proteins. The Tektin family of coiled-coil domain containing proteins was previously identified in cilia of organisms as diverse as green algae and sea urchin. While studies have shown that some Tektins are necessary for ciliary function, there has been no comprehensive phylogenetic survey of *tektin* genes. To fill this gap, we sampled *tektin* sequences broadly among metazoan and unicellular lineages in order to determine how the *tektin* gene complements evolved in over 100 different extant species.

**Results:**

Using Bayesian and Maximum Likelihood analyses, we have ascertained with high confidence that all metazoan *tektins* arose from a single ancestral *tektin* gene in the last common ancestor of metazoans and choanoflagellates. Gene duplications gave rise to two *tektin* genes in the metazoan ancestor, and a subsequent expansion to three and four *tektin* genes in early bilaterian ancestors. While all four *tektin* genes remained highly conserved in most deuterostome and spiralian species surveyed, most *tektin* genes in ecdysozoans are highly derived with extensive gene loss in several lineages including nematodes and some crustaceans. In addition, while *tektin-1, − 2,* and *− 4* have remained as single copy genes in most lineages, *tektin-3/*5 has been duplicated independently several times, notably at the base of the spiralian, vertebrate and hymenopteran (Ecdysozoa) clades.

**Conclusions:**

We provide a solid description of *tektin* evolution supporting one, two, three, and four ancestral *tektin* genes in a holozoan, metazoan, bilaterian, and nephrozoan ancestor, respectively. The isolated presence of *tektin* in a cryptophyte and a chlorophyte branch invokes events of horizontal gene transfer, and that the last common ciliated eukaryotic ancestor lacked a *tektin* gene. Reconstructing the evolutionary history of the *tektin* complement in each extant metazoan species enabled us to pinpoint lineage specific expansions and losses. Our analysis will help to direct future studies on Tektin function, and how gain and loss of *tektin* genes might have contributed to the evolution of various types of cilia and flagella.

**Electronic supplementary material:**

The online version of this article (10.1186/s12862-019-1360-0) contains supplementary material, which is available to authorized users.

## Background

Cilia and flagella are complex organelles found throughout the major domains of the tree of life [1] playing a wide variety of roles in locomotion and sensory functions. Some differences have been pointed out between cilia and flagella, such as that flagella are typically, but not always, longer than cilia, and cells may have anywhere from one primary cilium to numerous motile cilia, while cells are typically limited to one to a few flagella. Additionally, motile cilia and flagella differ in motility, with cilia beating in a stiff, oar-like power stroke, and flagella moving in a more whip-like, undulating fashion [2]. However, beyond these differences, cilia and flagella share a number of remarkable similarities. They share a similar internal structure, being supported by the axoneme, a cytoskeletal structure made up of microtubules arranged in a circular pattern of nine doublets and two central singlets. These provide both structural support and a means of movement, allowing cilia to perform diverse functions [3].

The presence of motile cilia and flagella as a means of providing motility in unicellular organisms such as the paramecium and the green algae *Chlamydomonas reinhardtii* indicate an ancient conserved role for these structures in providing cellular motility [1, 4]. This role is conserved in a variety of metazoan species as well, both in embryonic and adult stages. The placozoan *Trichoplax adhaerens* relies on a heavily ciliated epithelium for locomotion [5], as do the trochophore and lophophorate embryos and larvae of many spiralians such as the annelid *Platynereis dumerilii* [6, 7]. Several metazoans rely on motile cilia for locomotion in their adult form such as many species of gastropods [8, 9] and the flatworm *Schmidtea mediterranea* [10] which move about via a ciliated ventral epithelium, and ctenophores which rely on the coordinated beating of rows of long cilia, the combs, to swim [11]. In addition, most organisms with motile sperm rely on flagella for sperm locomotion, including some multicellular plants and green algae in which sperm is the only flagellated cell type produced in their life cycle [12–15], and most metazoans. Nematodes are a notable exception, as they produce amoeboid sperm [16]. In sponges a specialized cell type utilizes the beating of flagella to create fluid flow and actively pump water for filtration, removing food particles for phagocytosis. This cell type has been named ‘choanocytes’ due to their similarity in form and function to choanoflagellates, the group of unicellular organisms that is considered sister group to metazoans [17].

**Fig. 1:**
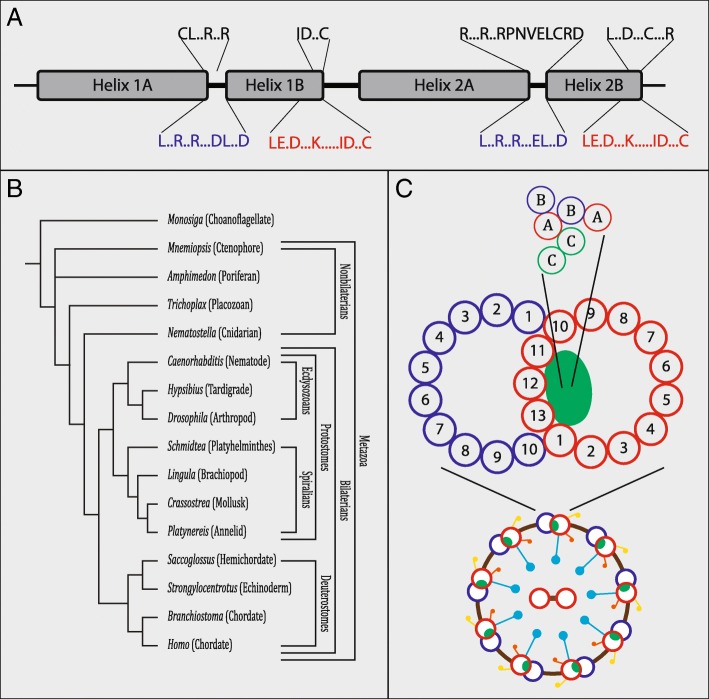
Structure and Localization of Tektins. **a** Schematic of the general structure of Tektin proteins consisting of two N-terminal alpha-helices (Helix 1A and Helix 1B) and a pair of C-terminal alpha-helices (Helix 2A and Helix 2B), separated by linker regions. Conserved amino acid motifs identified by Amos [20] are shown above. Conserved amino acid motifs identified in this study indicate similar motifs in the linker regions between the A and B helices of both alpha-helix pairs (black letters) as well as at the C-terminus of both B helices (red letters) are shown below. **b** Phylogenetic tree of metazoans and choanoflagellates indicating the evolutionary positions of the major phyla and key species examined in this study. **c** Location and composition of Tektin filaments within the axoneme of a motile cilium as proposed by Linck et al. [24]. The lower scheme shows a transverse section of the axoneme with the typical 9 plus 2 arrangements of 9 pairs of complete and incomplete microtubules (A in red, and B in blue, respectively) surrounding a central pair of A microtubules. Outer (yellow) and inner (red) dynein motor complexes, as well as radial spokes (blue) originating from each A microtubule are shown. The upper scheme details one doublet microtubule showing the thirteen and ten tubulin protofilaments that constitute the A- and B-microtubule, respectively, and the proposed localization of the Tektin filament (green sphere). Tektin filaments are thought to run along the inside of the A microtubule in each microtubule doublet of the axoneme. Tektin filaments are thought to be composed of multiples of three protein dimers: two Tektin-A/Tektin-B (Tektin-4/Tektin-2) heterodimers (thin red A and blue B circles) and one Tektin-C (Tektin-1) homodimer (thin green C circles)

In addition to microtubules, cilia and flagella are composed of many other proteins such as Nexins and Dyneins providing both structural support and motor functions, respectively [3]. Another family of proteins that have been implicated in cilia and flagellar structure and function are the Tektins, a family of coiled-coil domain containing proteins first isolated from sea urchin sperm flagella [18]. Tektin proteins have been discovered throughout many metazoan lineages as well as in unicellular organisms such as the algae *C. reinhardtii* [19]. Tektin proteins are primarily composed of long alpha helices encompassing the highly conserved Tektin domain (Fig. 1a) [20]. To date most Tektin studies have been carried out in mouse, rat and the sea urchin *S. purpuratus. Tektin* mutants have been found to cause defects in sperm flagella, often inhibiting fertility, as well as in cilia, affecting structure and motility. As many as five Tektins have been identified in vertebrates (Tektin-1 to Tektin-5) while in sea urchins Tektin-A, Tektin-B and Tektin-C, orthologous to Tektin-4, Tektin-2 and Tektin-1, respectively, [20] have been most extensively studied on a structural and biochemical level. Work in sea urchins has determined that Tektins form long filaments localized to the axoneme of cilia (Fig. 1c). Each filament is thought to be constituted of many subunits formed by three Tektin dimers: two dimers are composed by one Tektin-A and one Tektin-B molecule each forming two heterodimers, and a third dimer is formed by two Tektin-C molecules forming one homodimer [21, 22]. Thus, each filament is made up of multiples of these three dimers supported by the finding of a 1:1:1 ratio for Tektin-A, -B, and C-proteins in motile cilia [21, 23]. Recent work has determined that Tektin filaments are found within the A microtubule of each microtubule doublet and run along the length of the axoneme, and potential roles as ‘rulers’ to determine cilia length and stability have been suggested [21, 22, 24]. In mammals, mutations in *tektin-2*, *tektin-3* and *tektin-4* have led to sperm defects and subfertility [25–27]. Tektins may also play roles other than forming the Tektin filament. Additional studies on mammalian sperm have shown Tektin proteins localizing to different parts of the sperm, including Tektin-4 and Tektin-2 to the outer dense fibers [28, 29] and Tektin-5 to the mitochondrial sheath [30] of rodent sperm flagella. Tektin-1 has been shown to localize not only to the flagellum, but also to the acrosome of sperm in both bull and rodent [31]. In the urochordate, *C. intestinalis,* Tektin-1, − 2, and − 4 are found in both sperm flagellum and branchial cilia, while Tektin-3/5, which has not previously been shown to be part of the Tektin filament, is found only in branchial cilia but not sperm flagella [32]. With the exception of a handful of studies showing a connection between Tektin and flagella function in the algae *C. reinhardtii* [19], and expression studies in a few spiralians [33–35] and insects [36], very little is known about Tektin function and expression outside of mammals and echinoderms. In addition, very few phylogenetic analyses of Tektin proteins have been attempted. We have found only one previous attempt to analyze Tektin evolution [20], however, this study was hampered by limited data availability at the time, with only a single spiralian species and no metazoan species outside bilaterians included.

Here we have attempted the first comprehensive phylogenetic analysis of the *tektin* gene family utilizing Tektin protein sequences from every major metazoan clade, including recently available data from a number of spiralian, and nonbilaterian, as well as unicellular species. We provide a strongly supported framework of *tektin* evolution, elucidating not only the relationship of metazoan *tektins* with unicellular *tektins*, but also detailing the evolutionary history and diversification of *tektins* within 109 metazoan species and several unicellular species. Thus, this analysis captures the detailed description of expansion and contraction of the *tektin* gene complement in various metazoan lineages via evolutionary loss and gain of distinct *tektin* genes. This solid framework enables the formulation of new hypotheses about how changes to the *tektin* gene complement may have contributed to the evolution and diversity of ciliary and flagellar functions. Furthermore, it also suggests new avenues of research into the functional diversification of the *tektin* gene family by identifying species that have retained ancestral versus those species that exhibit a strongly modified *tektin* gene complement through duplications and losses. We suggest that the focus on key species with a defined *tektin* gene complement – ancestral or modified - will enable future studies to harness this diversity to answer fundamental questions about the role of Tektins in various cilia and flagella bearing cell types.

## Results

### Number of metazoan *tektin* genes in eukaryotic genomes

To elucidate the evolutionary history of the *tektin* gene family, we used reciprocal BLAST searches (see Methods) to survey 109 species from the major metazoan lineages with a preference for phylogenetically informative taxa along with 17 unicellular species (Additional file 1). At least one *tektin* sequence was identified in 111 different species including all representatives from all major metazoan phyla except Placozoa as well as two choanoflagellates and four other unicellular organisms from the Chlorophyta and Cryptophyta. We were unable to find *tektins* in any other unicellular eukaryotic lineages most of which possess motile cilia including other opisthokont phyla or alveolate ciliates (*Tetrahymena, Paramecium*) or euglenozoans (*Trypanosoma*). Some of these species were selected to investigate whether gain or loss of distinct *tektin* genes was species or clade specific. Table 1 and Additional file 1 show the number of *tektin* genes found in distinct eukaryotic and metazoan taxa and species. It ranges from none in the placozoan *Trichoplax adhaerens* to up to ten genes in the annelid leech *Helobdella robusta* and the planarian *Schmidtea mediterranea*, with the majority of bilaterian species containing 4 or 5 *tektin* genes, and prebilaterians like sponges and cnidarians two or three *tektin* genes, respectively. Outliers are the bilaterian nematode species and the crustacean *Daphnia pulex* that contain only one *tektin* gene, and the prebilaterian ctenophores that contain four genes. Interestingly, only four or five *tektin* genes are found in vertebrates, the same number as is average in bilaterian invertebrates. This is surprising because for many gene families the vertebrate gene complement is increased by up to a factor of four in comparison to the invertebrate bilaterian gene complement as a result of two well documented ancient whole genome duplication events at the base of the vertebrate lineage [37, 38].

**Table 1:**
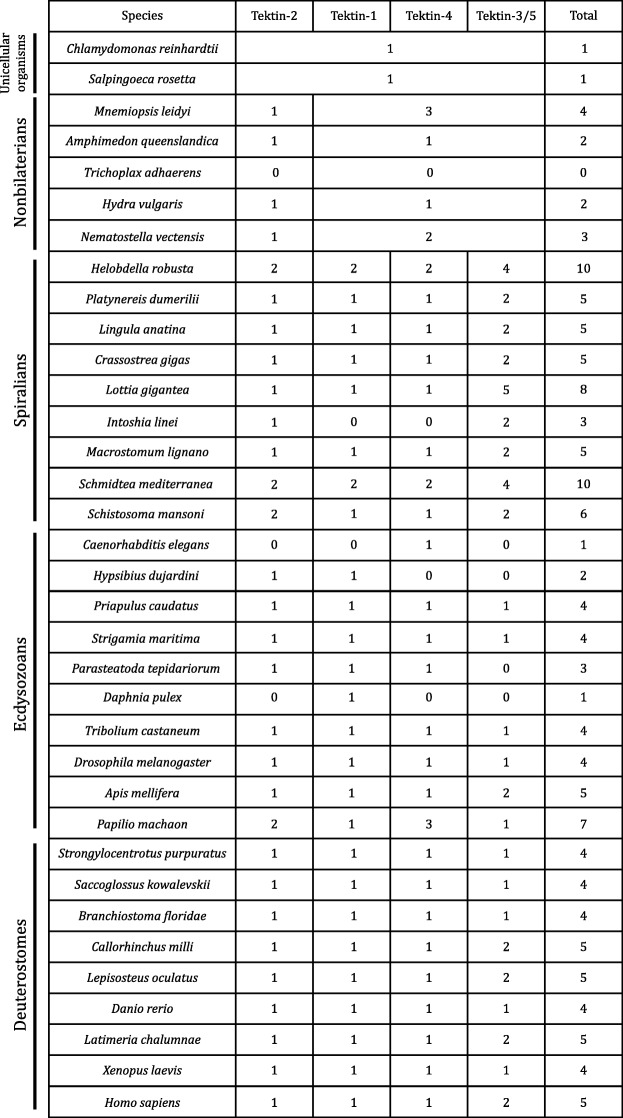
*Tektin* gene complement in metazoan species

The table shows selected species of all major lineages examined in this study and their *tektin* gene complement. Species are listed according to five groups indicated on the left: Unicellular organisms, nonbilaterians, spiralians, ecdysozoans, and deuterostomes. The *tektin* gene complement for each species is presented by total number of *tektin* genes within their genome in the right column, as well as the number of homologs for each of the four bilaterian classes: Tektin-2, Tektin-1, Tektin-4, and Tektin-3/5. Note that these four Tektin classes are bilaterian specific originating by ancient gene duplications from two nonbilaterian and one unicellular *tektin* gene(s). The number of *tektin* genes in species range from zero in placozoans up to ten in the leech and planarian, and demonstrate substantial gain and loss of *tektin* genes in various metazoan lineages. A complete table of all species included in this study, with the exception of some teleost fish with identical *tektin* complements, is shown in Additional file 1

### Structure of metazoan Tektin proteins

**Fig. 2:**
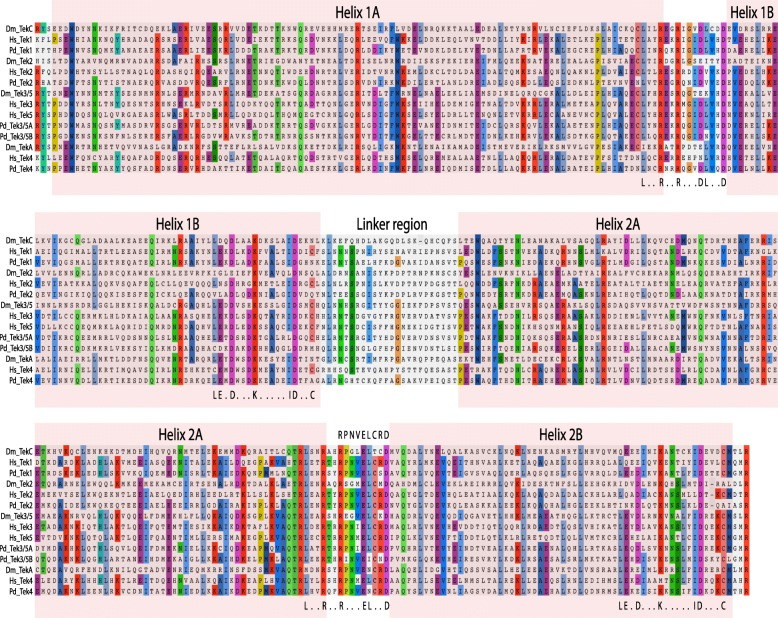
Alignment and conserved motifs of Tektin proteins. Amino acid alignment of conserved regions of the Tektin protein complements from *D. melanogaster (Dm), H. sapiens (Hs)* and *P. dumerilii (Pd)* are shown grouped by the four Tektin classes: Tektin-1 or C, Tektin-2, Tektin 3/5, and Tektin-4 or A*.* The Tektin-3/5 class includes two human (3 and 5) and two *Platynereis* (3/5A and 3/5B) Tektins. Two pairs of alpha helices (Helix 1 and 2) are highlighted by pink boxes (compare to Fig. 1a). Conserved residues are color-coded using Aliview. The highly conserved nonapeptide motif between Helix 2A and Helix 2B [20] is highlighted above the alignment. Motifs identified in this study are highlighted below the alignment, and include a highly conserved motif (L . . R . . R . . . D/E L . . D) within the short regions between Helix 1A and 1B and Helix 2A and 2B, and a second conserved motif (L E . D . . . K . . . . . I D . . (.) C) within the C-terminus of Helix 1B and Helix 2B

The Tektin proteins that we surveyed were typically 400 to 450 amino acids (aa) in length for Tektin-1, and 400 to 550 aa for Tektins-2, − 4, and − 3/5 (Figs. 1a, 2 and Additional file 2). Some sequences were found to be significantly shorter than 400 aa, but we assume these are partial sequences, while a few sequences significantly longer than 550 aa were likely due to assembly artifacts. Previous studies indicate that Tektin proteins are composed of an extended coiled-coil domain approximately 380 aa in length [20, 21, 39]. This central domain is typically flanked by less conserved N- and C-terminal regions. Located in the middle of the conserved coiled-coil domain is a characteristically less conserved linker region separating the alpha-helices in the N-terminal half from the alpha-helices in the C-terminal half of the domain [20]. The conserved region of the Tektins generally share ~ 40–60% identity with other Tektins of the same class, and ~ 20–45% identity with Tektins of a different class (Additional file 3). The conserved coiled-coil domain is subdivided into four alpha helix domains: Helix 1A and Helix 1B at the N-terminus, and Helix 2A and Helix 2B at the C-terminus. In addition to the nonapeptide motif between Helix 2A and Helix 2B identified by Amos et al. [20], we identified a highly conserved LxxRxxRxxxE/DLxxD motif found both between Helix 1A and 1B and between Helix 2A and 2B. An additional conserved motif, LexDxxxKxxxxxIDxx(x)C, occurs at the C-terminal end of Helix 1B and 2B (Figs. 1a and 2). For our analysis, we used an alignment of 385 aa that covered the conserved coiled-coil domain and conserved flanking residues on either end (Additional file 4).

### Phylogenetic analyses of the *tektin* gene complements

To date the evolutionary history of *tektins* is largely unknown, especially outside the deuterostomes, due to a lack of comprehensive phylogenetic analyses. To fill this gap, we attempt (1) to assign bilaterian *tektins* to distinct classes, (2) to identify their orthologs in the nonbilaterians and unicellular organisms, and (3) to delineate the origin of each extant metazoan *tektin* gene. Furthermore, we aim (4) to identify species or clades that retain ancestral *tektin* gene complements or have undergone gene gain and/or gene loss. This information will be helpful for identifying metazoan species for future studies that could elucidate the roles that *tektins* have played in shaping cilia and flagella evolution and diversification. For terminology, we utilize the naming convention for the vertebrate/mammalian *tektin* gene complement (*tektin-1, tektin-2, tektin-3, tektin-4, tektin-5*).

Within metazoans we collected sequences from three ctenophores, seven poriferans, six cnidarians, four xenacoelomorphs, 31 spiralians, 31 ecdysozoans and 24 deuterostomes, but were unable to find *tektins* in the single placozoan species (*Trichoplax*) (see Additional file 5 for complete list). We performed two phylogenetic analyses for each data set: maximum likelihood and Bayesian inference. For maximum likelihood we ran 1000 bootstraps of the program RAxML [40] using LG model of substitution rates with gamma parameter and a proportion of invariant sites. For Bayesian inference we used the program Mr. Bayes [41]. Analysis was run for 2,000,000 generations with a burn in of 500,000 using the mixed model of substitution rates with gamma parameter and a proportion of invariant sites. For further details of alignment and phylogenetic analyses see Methods. Very long branched taxa and partial sequences were removed from final analyses. Various phylogenetic analyses enabled us to suggest a conclusive evolutionary scenario for the *tektin* gene complements (I) within metazoans, (II) within nonbilaterians, (III) within bilaterians, and for each of the four ancestral bilaterian *tektin* genes, (IV) *tektin-2*, (V) *tektin-1*, (VI) *tektin-4*, and (VII) *tektin-3/5.*

### I. Overview: The emergence of the *tektin* gene complement in metazoans

**Fig. 3:**
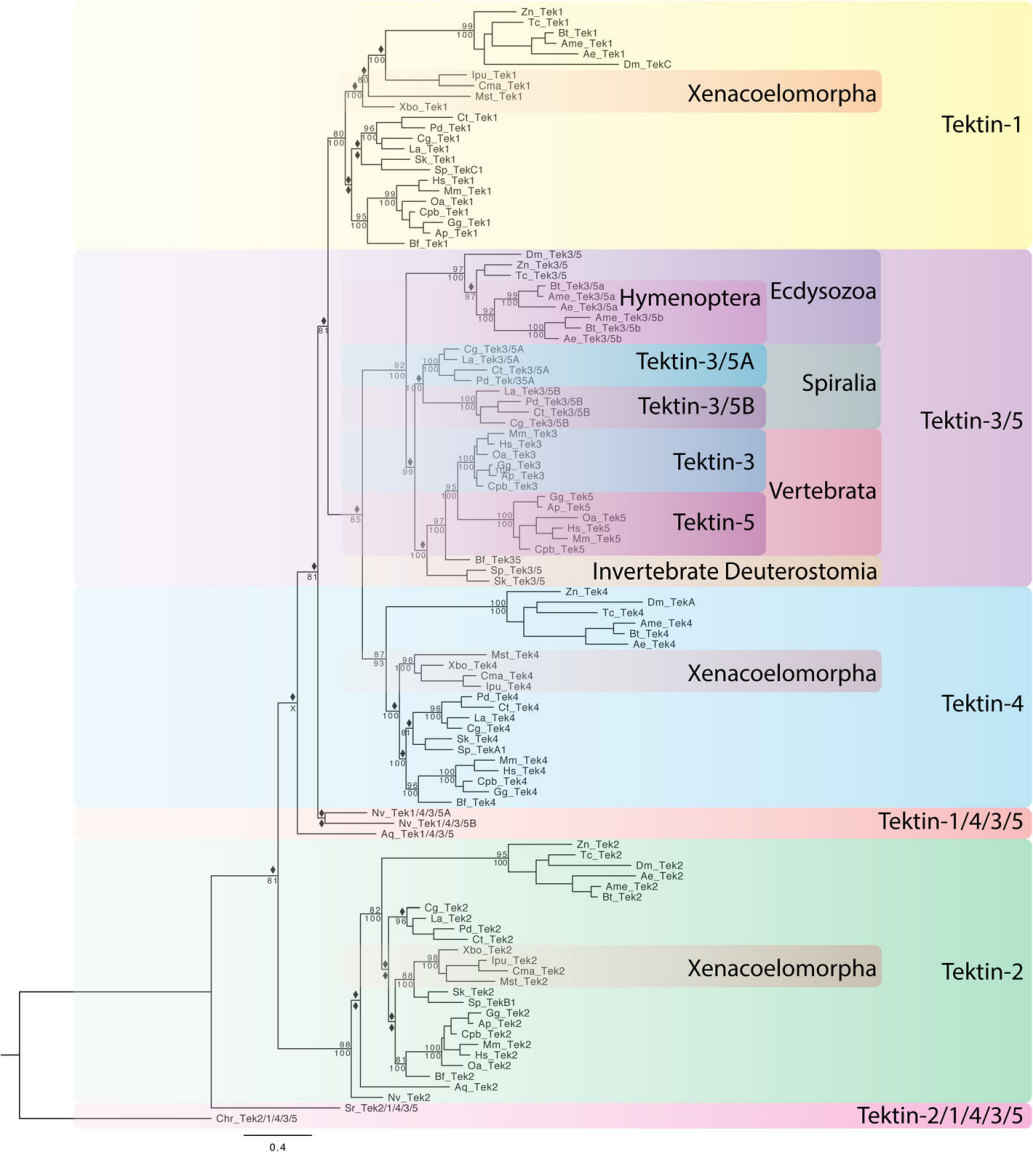
Phylogenetic tree to illustrate the evolution of the Tektin complement in metazoan species. This phylogenetic analysis includes the full Tektin complements identified in selected metazoan species including four xenacoelamorphans (Cma, Ipu, Mst, Xbo), six ecdysozoans (Ae, Ame, Bt, Dm, Tc, Zn), four spiralian species (Cg, Ct, La, Pd), three invertebrate deuterostomes (Sk, Sp, Bf), and six vertebrate deuterostomes (Ap, Cpb, Gg, Mm, Oa, Hs), as well as two nonbilaterians (Aq, Nv), one choanoflagellate (Sr) and a green algae (Chr) as an outgroup. Both Bayesian and Maximum Likelihood analyses were performed using Mr. Bayes and RAxML, respectively. The Bayesian tree topology is shown. Node support is shown for non-terminal nodes. Posterior probability values from Mr. Bayes and bootstrap values from RAxML are shown above or below each node, respectively. Diamonds indicate support less than 80%. “X” under a node indicates that this node was not recovered in the RAxML maximum likelihood tree. Tree was rooted with green algae (Chr) Tektin. The large colored boxes highlight the four major Tektin classes that exist in bilaterians, Tektin-2 (green), Tektin-4 (light blue), Tektin-1 (yellow), and Tektin-3/5 (purple), the nonbilaterian class Tektin-1/4/3/5 (orange) and the ancestral non-metazoan Tektin-2/1/4/3/5 (pink). The smaller colored boxes within the Tektin-3/5 class group Tektins that belong to higher taxa as indicated to highlight the three independent duplications of Tektin-3/5 at the base of the ecdysozoan hymenopterans (Tektin-3/5a and − 3/5b), the spiralians (Tektin-3/5A and 3/5B), and the deuterostome vertebrates (Tektin-3 and Tektin-5). All metazoans share Tektin-2 while the nonbilaterian Tektin-1/4/3/5 is sister group to the three exclusively bilaterian Tektin classes Tektin-1, Tektin-4, and Tektin-3/5. Tektins are named according to the groups recovered by this phylogenetic analysis. Species abbreviations and accession numbers for each sequence are provided in Additional file 5

Various phylogenetic analyses of the *tektin* gene complements, the distribution of *tektin* genes within each species and animal lineages, in comparison to the well-established general animal phylogeny suggest three key transitions in the emergence of the *tektin* complement within metazoans. Although alternative evolutionary scenarios are possible, we consider these key transitions as most likely (Fig. 3 and Additional file 6; see also Fig. 9a): (1) All extant metazoan *tektins* arose via duplication from a single ancestral *tektin* gene that was present in the last common ancestor of metazoans and choanoflagellates. The evolutionary relationship to the only other tektins found outside holozoans in a few isolated unicellular organisms, a cryptophyte and a small branch of chlorophytes, is unclear but may suggest two events of horizontal gene transfer between these very distantly related eukaryotic branches. We were unable to identify any unicellular organisms that possess more than one *tektin* gene. We have designated this ancestral *tektin*, still retained in single copy in the holozoan choanoflagellates, *tektin-2/1/4/3/5* to indicate its homology to all metazoan *tektins*. (2) According to our analysis there was a duplication event early in the metazoan lineage prior to the divergence of ctenophores and poriferans from the remainder of the metazoans. This gave rise to two putative ancestral *tektins* that we have named *tektin-2* and *tektin-1/4/3/5* to indicate their homology to extant bilaterian *tektins*. Of the 17 nonbilaterian metazoan species that were surveyed, the *tektin* complement ranged from zero in the placozoan *T. adhaerens* to four in ctenophores (Table 1 and Additional file 1). None of the nonbilaterian species that we analyzed had more than one copy of *tektin-2*. All species with more than two *tektin* genes appear to have undergone duplications of the *tektin-1/4/3/5* gene. Thus, all prebilaterian metazoan *tektin* genes most likely arose from two ancestral genes *tektin-2* and *tektin 1/4/3/5*. (3) All extant bilaterian Tektins can be assigned to one of four classes originating from one of four ancestral *tektin* genes, *tektin-2, tektin-1, tektin-4,* and *tektin-3/5.* Bilaterian Tektin-2 groups strongly with the nonbilaterian Tektin-2 s. Bilaterian Tektin-1, Tektin-4 and Tektin-3/5 all group strongly within the nonbilaterian Tektin-1/4/3/5 s. Thus, despite the fact that some nonbilaterians possess multiple copies of Tektin-1/4/3/5, our analysis indicates that all extant bilaterian *tektins-1, − 4,* and *− 3/5* arose via duplication from a single ancestral *tektin-1/4/3/5* gene.

### II. Tektin evolution within nonbilaterian lineages

Our analysis is most consistent with a scenario in which all metazoan *tektins* arose via duplication of a single ancestral *tektin 2/1/4/3/5* gene that was present in a unicellular holozoan ancestor. We have identified six unicellular species that exhibit this ancestral state of a single *tektin* gene. Surprisingly, these include four algae species, the cryptophyte *G. theta* and three chlorophytes – *V. carteri, G. pectorale* and *C. reinhardtii*, as well as two choanoflagellates, *S. rosetta* and *M. ovata.* Thus, the ancestral state of a single *tektin* has been retained without any indication for any persisting gene duplicates in these unicellular eukaryotes. Curiously, we did not find any putative *tektins* in other unicellular eukaryotic lineages that all possess motile cilia and/or flagella including members of the phyla Ciliophora or Dinoflagellata, or the genus Plasmodia (Table 1 and Additional file 1). Thus, the presence of a Tektin homolog in only a few algal species and the very distantly related holozoans is most likely the result of horizontal gene transfer. The alternative scenario, an ancestral *tektin* gene inherited from the last common eukaryotic ancestor, would have required many independent losses of *tektin* genes in over a dozen of eukaryotic branches.

**Fig. 4:**
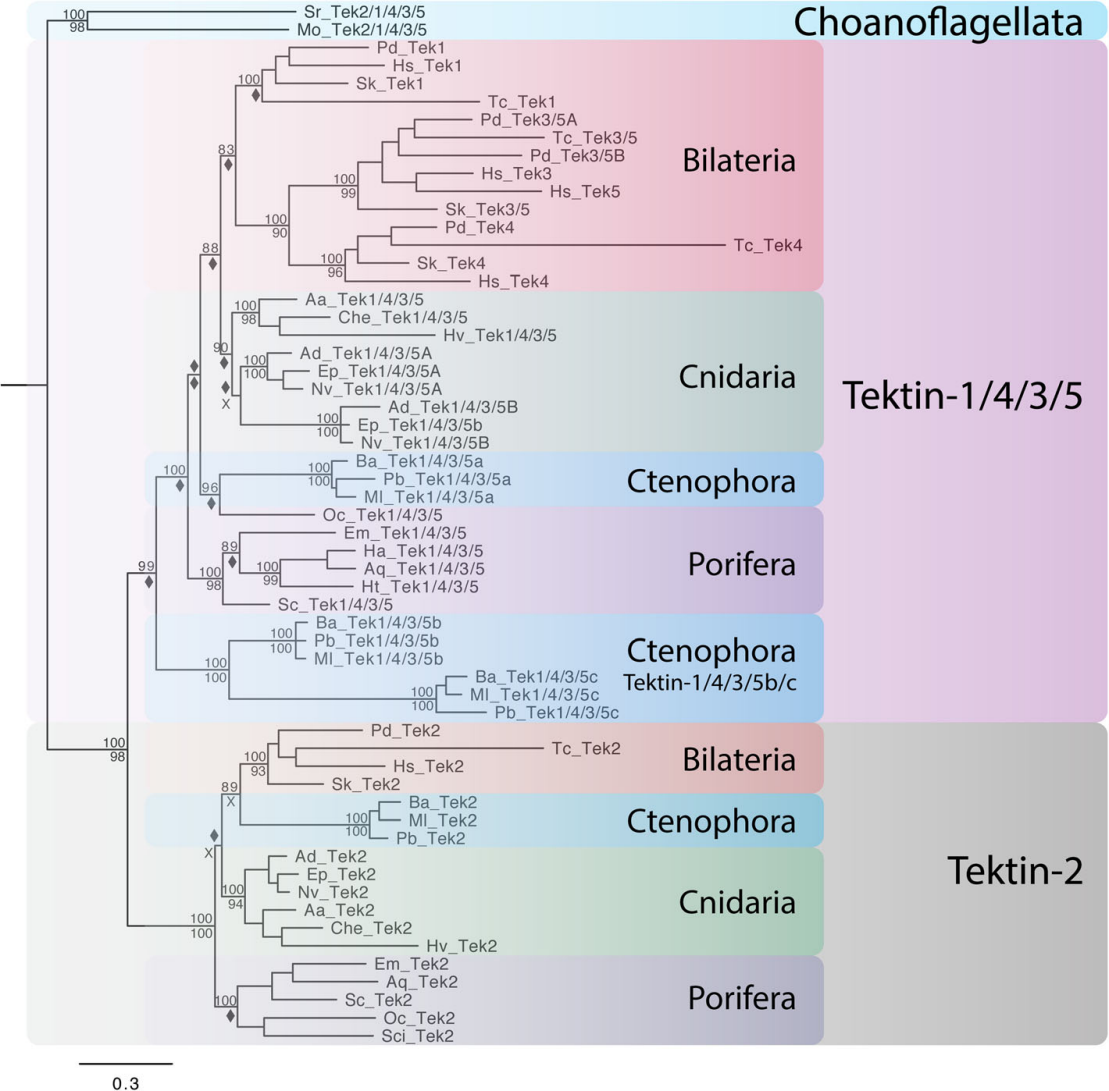
Phylogenetic tree to illustrate the evolution of the Tektin complement in nonbilaterian species. This phylogenetic analysis includes the full Tektin complements identified in six cnidarians (Aa, Che, Hv, Ad, Ep, Nv), three ctenophores (Ba, Pb, Ml), six poriferans (Oc, Em, Ha, Aq, Ht, Sc), and four bilaterians (Tc, Pd, Hs, Sk), as well as two choanoflagellates (Sr and Mo) as outgroups. Both Bayesian and Maximum Likelihood analyses were performed using Mr. Bayes and RAxML, respectively. The Bayesian tree topology is shown. Node support is shown for non-terminal nodes. Posterior probability values from Mr. Bayes and bootstrap values from RAxML are shown above or below each node, respectively. Diamonds indicate support less than 80%. “X” under a node indicates that this node was not recovered in the RAxML maximum likelihood tree. Tree was rooted with choanoflagellate sequences. Nonbilaterian Tektins fall into one of two groups: Tektin-2 and Tektin-1/4/3/5. Tektins are named according to the groups recovered by this phylogenetic analysis. Species abbreviations and accession numbers for each sequence are provided in Additional file 5

Metazoan nonbilaterian taxa comprise four main phyla, the sponges (Porifera), the comb jellies (Ctenophora), the single species phylum Placozoa, and the corals and jellyfish (Cnidaria) with a currently contended phylogenetic branching pattern (Fig. 1b). Most contentious is the question of which phyla is the sister group to the remaining Metazoa – Porifera or Ctenophora [42–46]. In contrast, Cnidaria are well established as the sister group to Bilateria. Interestingly, our phylogenetic analysis of Tektin proteins supports a clear distinction between the nonbilaterian versus the bilaterian taxa suggesting that the ancestral nonbilaterian *tektin* gene complement comprised of two *tektin* genes, and the ancestral bilaterian of four (Figs. 3, 4, and Additional file 6). Thus, there were two *tektins* present in the last common ancestor of the metazoans: *tektin-2* and *tektin-1/4/3/5*, and our analysis suggests that these two *tektin* genes gave rise independently to the extant *tektin* complement in Porifera (1 to 2 genes), Ctenophora (4 genes), Placozoa (0 gene), Cnidaria (2 to 3 genes) and Bilateria (4 ancestral genes).

Of the seven poriferan species surveyed, five retain this ancestral state of two *tektins*, while two demosponge species from the genus *Haliclona* retain only one, having presumably lost *tektin-2*. We surveyed three ctenophore species, each of which possesses four *tektin* genes that are orthologous to each other suggesting a set of four distinct ancestral ctenophore *tektin* genes. Of these four, one groups unambiguously with the poriferan and other *tektin-2 s*, and was named ctenophore *tektin-2*. While one other ctenophore *tektin* groups clearly with poriferan and other *tektin-1/4/3/5 s*, the two remaining form a cluster basal to all *tektin-1/4/3/5 s* (Fig. 4). As several analyses have placed the ctenophores as the sister group to all other metazoans [42, 45], this could indicate a second duplication event prior to divergence of the ctenophores from the rest of the metazoans followed by a deletion in the ancestor of poriferans and cnidarians. Alternatively, and consistent with more recent studies that support a more basal position for the sponges [44, 46]*,* this duplication took place specifically in the early ctenophore lineage after their divergence from other metazoans, and the basal position of this ctenophore tektin branch might be due to long-branch attraction. Therefore, we suggest that the extant ctenophore complement of four *tektins* arose from two independent duplications of the ancestral metazoan *tektin-1/4/3/5* gene in the early ctenophore lineage (ctenophore Tektin-1/4/3/5a, −b, and -c), and the retention of an ancestral *tektin-2* gene (ctenophore Tektin-2). Interestingly, no *tektin* gene was detected in the single placozoan species *Trichoplax* suggesting the loss of two ancestral metazoan *tektin* genes in this placozoan (Table 1 and Additional file 1).

Although our survey was limited to six cnidarian species only, our analysis found evidence for the retention of the ancestral metazoan *tektin-2* gene and at least one *tektin-1/4/3/5* gene in each cnidarian species. While the two hydrozoan species *H. vulgata* and *C. hemisphaerica* retain single genes for each of the two ancestral Tektins, three anthozoan and one scyphozoan species possess three orthologous *tektin* genes, indicating an additional independent duplication of *tektin-1/4/3/5* at some point in the cnidarian lineage. Thus, although the main target of this study was to ascertain the evolutionary relationships of bilaterian *tektins* our phylogenetic analyses provides a solid hypothesis for pre-bilaterian metazoan *tektin* evolution, that should be further tested by broader sampling.

### III. The emergence of the bilaterian *tektin* gene complement

As described above our analyses strongly support an evolutionary scenario that suggest that four distinct bilaterian *Tektin* classes 2, 1, 4, and 3/5 arose from two ancestral *tektin* genes present in the last common ancestor of bilaterians and cnidarians: *tektin-2 and tektin-1/4/3/5* (Figs. 3, 9a, Additional file 6). Here, we summarize some of the general observations about the emergence and evolutionary trajectory of the four bilaterian *tektin* genes: Intriguingly, the bilaterian *tektin-2* gene remained a single copy gene in nearly all 87 bilaterian species surveyed, while losses or duplications of this gene are very rare. In contrast, the ancestral metazoan *tektin-1/4/3/5* gene underwent two duplication events prior to the protostome/deuterostome split. Our phylogenetic analysis suggests that the first duplication gave rise to the ancestral bilaterian *tektin-1* and a proto-*tektin-4/3/5* gene. This ancestral *tektin-4/3/5* then underwent a second duplication to give rise to the two ancestral *tektin-4* and *tektin-3/5* genes. This duplication may have occurred after the divergence of the basal bilaterian phyla Xenacoelomorpha from the protostomes/deuterostome lineage. Intriguingly all four Xenacoelomorpha species surveyed appear to lack *tektin-3/5,* but retain a clear *tektin-4* homolog. Although this Xenacoelomorpha *tektin* gene clusters unambiguously with other *tektin-4* s in both maximum likelihood and Bayesian analyses, it actually might represent an early bilaterian *tektin-4/3/5* gene which groups with *tektin-4* due to the higher sequence divergence of protostome and deuterostome *tektin-3/5 s*. Thus, these four *tektin* classes 2, 1, 4 and 3/5 were present before the protostomes and deuterostomes, and *tektin-3/5* might be an ancestral nephrozoan tektin gene rather than an ancestral bilaterian one.

Intriguingly, similar to the *tektin-2* gene, both the *tektin-1* and *tektin-4* genes remained single copy genes in most bilaterian species exhibiting only rarely a duplication event (see Discussion below; Table 1 and Additional file 1). This is especially surprising within the vertebrate lineage that experienced two rounds of whole genome duplication after branching from the chordate ancestor [37, 38]. Thus, any gene duplicates of these three *tektin* genes that arose from these ancient genome duplications must have been rapidly lost. In contrast to the evolutionary ‘stasis’ of these three ancestral *tektin* genes within bilateria, we found evidence for several independent duplications of the latest emerged ancestral *tektin-3/5* gene in each of the three major bilaterian lineages (see VII). One such gene duplication occurred early in the vertebrate lineage, likely due to the whole genome duplications, giving rise to the vertebrate specific *tektin-3* and *tektin-5* genes. Thus, while the last common ancestor of deuterostomes had four distinct *tektin* genes, as retained in all surveyed invertebrate deuterostomes, the last common ancestor of vertebrates already had five *tektin* genes.

Another independent duplication of the *tektin-3/5* gene occurred early in the spiralian lineage prior to its diversification, giving rise to two ancestral spiralian *tektin-3/5* genes that we have named *tektin-3/5A* and *tektin-3/5B* to distinguish them from vertebrate *tektin-3* and *tektin-5*. Therefore, we hypothesize that the last common ancestor of the spiralians included in our study already had five *tektin* genes also.

While the vast majority of the 31 ecdysozoan species we surveyed retain only a single *tektin-3/5* gene or none, we found evidence for a third independent *tektin-3/5* gene duplication specific to the hymenopteran branch (wasps, bees, ants) within the arthropod insects, giving rise to the hymenopteran specific *tektin-3/5a* and *tektin-3/5b*. Thus, while the last common ancestor of ecdysozoans had four *tektins*, the last common ancestor of the surveyed hymenopterans had five. An additional curious general observation of bilaterian *tektin* evolution is a highly increased divergence of Tektin protein sequences for the three ecdysozoans *tektin-2, − 1 and − 4*, but not *− 3/5* genes compared to orthologous spiralian and deuterostome *tektins*. This concerted divergence of the three ecdysozoan *tektin* genes is apparent by the longer branch lengths for nearly all ecdysozoan species (Fig. 3) and a much lower identity shared with orthologs (Additional file 3) suggesting a simultaneous event to relax the constraints on each of these three *tektin* sequences at the base of the ecdysozoan branch while maintaining strong constraints for spiralian and deuterostome *tektins*.

### IV. Origin and evolution of the bilaterian *tektin-2* gene

**Fig. 5:**
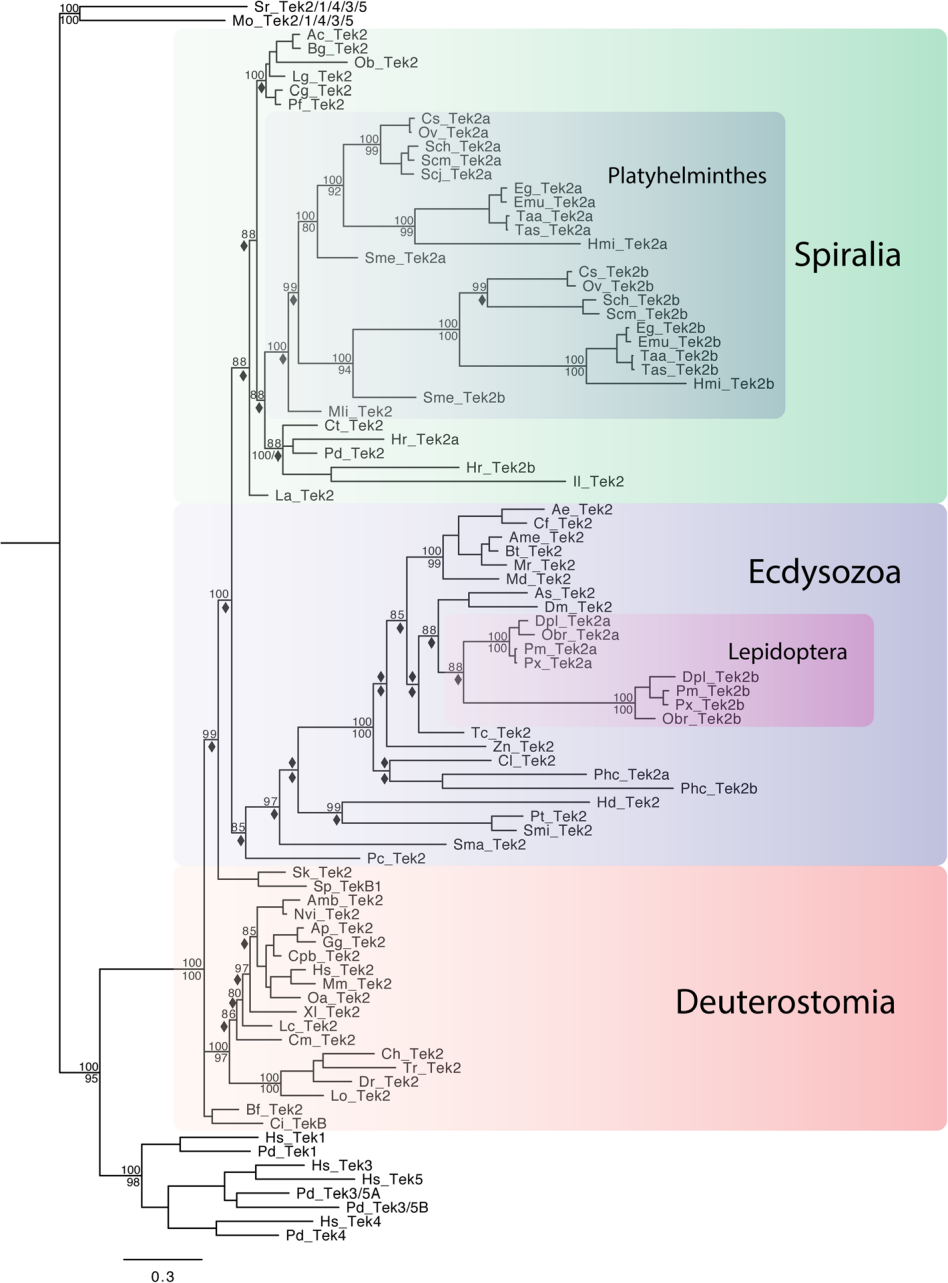
Phylogenetic tree to illustrate the evolution of Tektin-2 in bilaterian species. This phylogenetic analysis includes the Tektin-2 sequences identified in bilaterian species. The 23 spiralians include six mollusks (Ac, Bg, Ob, Lg, Cg, Pf), twelve platyhelminthes (Cs, Ov, Sch, Scm, Scj, Eg, Emu, Taa, Tas. Hmi, Sme, and Mli), three annelids (Pd, Ct, Hr), one brachiopod (La), and one orthonectid (Il). The ecdysozoans include many insects, two chelicerates (Pt, Smi), one myriapod (Sma), one tardigrade (Hd), and one priapulid (Pc). The invertebrate deuterostomes are represented by one echinoderm (Sp), one hemichordate (Sk), one cephalochordate (Bf), and one urochordate (Ci). The vertebrate deuterostomes are represented by three teleost fishes (Dr, Tr, and Ch), one holostei fish (Lo), one chondrichthyes (Cm), one sarcopterygian fish (Lc), three amphibians (Xl, Amb, Nvi), one reptile (Cpb), two avians (Ap, Gg), and three mammal species (Oa, Mm, Hs). In addition, the entire Tektin complement for an annelid (Pd) and human (Hs) was included as well as two choanoflagellate (Sr, Mo) sequences as outgroups. Both Bayesian and Maximum Likelihood analyses were performed using Mr. Bayes and RAxML, respectively. The Bayesian tree topology is shown. Node support is shown for non-terminal nodes. Posterior probability values from Mr. Bayes and bootstrap values from RAxML are shown above or below each node, respectively. Diamonds indicate support less than 80%. “X” under a node indicates that this node was not recovered in the RAxML maximum likelihood tree. The large colored boxes group Tektin-2 sequences of the three major bilaterian branches, the spiralians (green), the ecdysozoans (blue), and the deuterostomes (orange). The smaller colored boxes within ecdysozoans and spiralians highlight Tektin-2 gene duplications at the base of insect lepidopterans (light purple), and one duplication in the Platyhelminthes lineage including the planarian (Sme) and the parasitic trematodes and cestodes, but excluding the flatworm (Mli) (dark green), respectively. Note that *tektin-2* exists as a single copy gene in most extant species with only two additional duplicates observed in the leech (Hr) and louse (Phc). Species abbreviations and accession numbers for each sequence are provided in Additional file 5

As discussed above *tektin-2* represents an ancestral metazoan *tektin* gene that appears to have persisted as a single copy gene in most metazoan species including most bilaterians. While a few lineages such as nematodes and the crustacean *Daphnia pulex* appear to have lost *tektin-2*, all other deuterostomes, many spiralians and nearly all arthropods that we surveyed maintained a single *tektin-2*, which is orthologous to the *tektin-2* that we found in nonbilaterian lineages such as ctenophores, poriferans and cnidarians (Fig. 5, Table 1). Only two insect orders, most spiralian Platyhelminthes, and the leech *Helobdella robusta*, among all bilaterians surveyed, exhibit a duplication of *tektin-2* (Fig. 5, Table 1, Additional files 1, 6, 7). The representatives of the two insect orders are the anopluran *P. humanus corporis,* and four species of lepidopterans indicating a *tektin-2* gene duplication basally within the latter insect order. Because anoplurans and lepidopterans are not closely related and other insect orders have only a single *tektin-2,* these are most likely independent duplications. Interestingly, ten of eleven platyhelminthes species surveyed maintained two orthologous yet highly divergent *tektin-2* genes, named *tektin-2a* and *-2b* while the more basally branching Platyhelminthes *Macrostomum lignano* and *Prostheceraeus vittatus* each have only one highly conserved *tektin-2* gene (Additional file 7). This suggests a *tektin-2* gene duplication followed by subsequent sequence divergence sometime after the split of the Macrostomorpha, but before the divergence of the Adiaphanida, which includes *S. mediterranea*, and the parasitic Neodermata, which includes the cestodes and trematodes [47]. With the exception of two highly derived spiralian species, the orthonectid *I. linei* and the leech *H. robusta*, the remaining surveyed spiralians including the mollusks, annelids, and brachiopod share high sequence identity (~ 50%) and exhibit short branches compared to deuterostome *tektin-2 s* (Additional file 7). In contrast, ecdysozoan *tektin-2 s* including insects as well as the priapulid *P. caudatus* have universally long branches and low sequence identity (mid-30%) (Additional file 3) when compared to spiralians and deuterostomes, Thus, our analysis indicates that many bilaterian lineages exhibit a general high constraint preventing *tektin-2* gene duplication and counteracting sequence divergence (deuterostomes, and many spiralians), while others including most platyhelminthes and ecdysozoans exhibit a relaxation of these evolutionary constraints.

### V. Origin and evolution of the bilaterian *tektin-1* gene

**Fig. 6:**
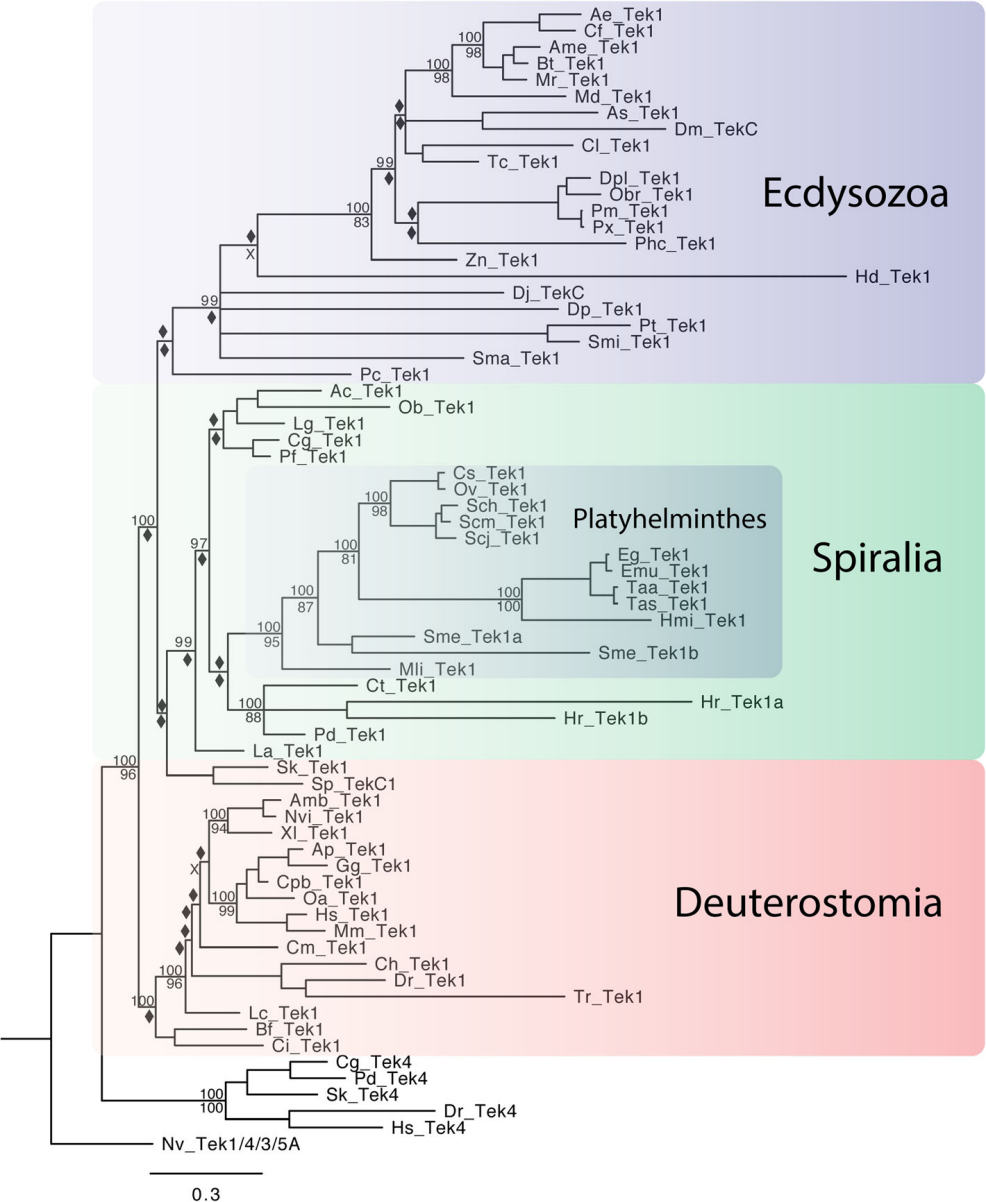
Phylogenetic tree to illustrate the evolution of Tektin-1 in bilaterian species. This phylogenetic analysis includes the Tektin-1 sequences identified in bilaterian species. The 23 spiralians include six mollusks (Ac, Bg, Ob, Lg, Cg, Pf), 12 platyhelminthes (Cs, Ov, Sch, Scm, Scj, Eg, Emu, Taa, Tas. Hmi, Sme, and Mli), three annelids (Pd, Ct, Hr), and one brachiopod (La). The ecdysozoans include many insects, two chelicerates (Pt, Smi), one myriapod (Sma), one crustacean (Dp), one dicyemid (Dj), one tardigrade (Hd), and one priapulid (Pc). The invertebrate deuterostomes are represented by one echinoderm (Sp), one hemichordate (Sk), one cephalochordate (Bf), and one urochordate (Ci). The vertebrate deuterostomes are represented by three teleost fish (Dr, Tr, and Ch), one holostei fish (Lo), one chondrichthyes (Cm), one sarcopterygian fish (Lc), three amphibian (Xl, Amb, Nvi), one reptile (Cpb) two avians (Ap, Gg), and three mammal species (Oa, Mm, Hs). In addition, Tektin-4 sequences from *C. gigas, P. dumerilii, S. kowalevskii, D. rerio* and *H. sapiens* were included as an outgroup. The tree was rooted with *N. vectensis* Tektin-1/4/3/5A. Both Bayesian and Maximum Likelihood analyses were performed using Mr. Bayes and RAxML, respectively. The Bayesian tree topology is shown. Node support is shown for non-terminal nodes. Posterior probability values from Mr. Bayes and bootstrap values from RAxML are shown above or below each node, respectively. Diamonds indicate support less than 80%. “X” under a node indicates that this node was not recovered in the RAxML maximum likelihood tree. The large colored boxes group Tektin-1 sequences of the three major bilaterian branches, the spiralians (green), the ecdysozoans (blue), and the deuterostomes (orange). Note that *tektin-1* exists as a single copy gene in most extant species with only two duplicate *tektin-1* genes observed in two spiralian species, a leech (Hr) and a planarian (Sme). Species abbreviations and accession numbers for each sequence are provided in Additional file 5

According to our analysis, the ancestral bilaterian *tektin-1*, *tektin-4* and *tektin-3/5* genes all arose from a single ancestral metazoan *tektin-1/4/3/5* gene. We hypothesize that an early duplication event of the te*ktin-1/4/3/5* gene in the bilaterian lineage gave rise to *tektin-1* and a *proto-tektin-4/3/5*. With the exception of nematodes and the spiralian orthonectid *I. linei* all bilaterian species we surveyed have at least one *tektin-1* gene, including the crustacean *D. pulex* that retains only *tektin-1* and no other *tektins* (Fig. 6, Table 1, Additional files 1, 6, 7 and 8). Only two bilaterian species, the spiralian leech *H. robusta* and the flatworm *S. mediterranea* have duplicated the *tektin-1* gene independently and exhibit high sequence divergence (Additional file 7). As observed for *tektin-2*, *tektin-1 s* in deuterostome and most spiralians – with the exception of ten of eleven platyhelminthes species - generally share high aa sequence identity (~mid-50%) and generally short branch lengths. In contrast, most ecdysozoan *tektin-1 s* are far more derived compared to their conserved spiralian and deuterostome counterparts, sharing less than ~ 40% (Additional file 3) identity with spiralian and deuterostome *tektin-1 s.* Ecdysozoan branch lengths for *tektin-1* are much longer than deuterostome and most spiralian branch lengths. Notable exceptions within their respective taxa are *tektin-1* of the ecdysozoan priapulid *P. caudatus,* and *tektin − 1* of the platyhelminthes *M. lignano* both of which are well conserved. In contrast, *tektin-1* appears highly conserved with short branch lengths and high sequence identity in deuterostome invertebrates (echinoderm, hemichordate, urochordate, and cephalochordate), and most spiralian species (brachiopod, mollusks, annelids but not leech). In summary, the ancestral bilaterian *tektin-1* gene exists as a single copy gene in most bilaterians. Loss, duplications and strong sequence divergence of *tektin-1* are observed but rare, and found in taxa known for their increased genomic divergence including the ecdysozoan arthropods and nematodes, and the spiralian platyhelminthes and leech.

### VI. Origin and evolution of the bilaterian *tektin-4* gene

**Fig. 7:**
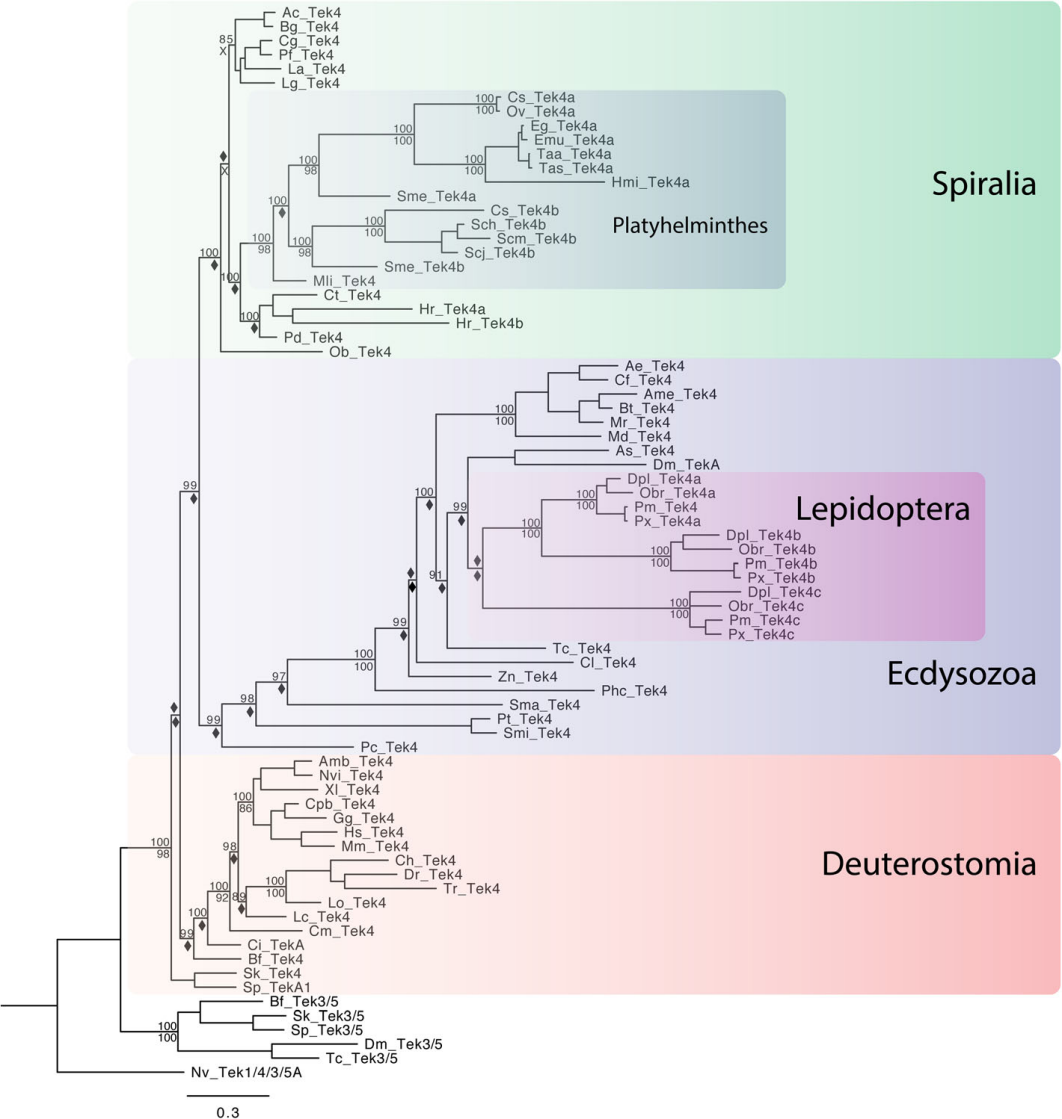
Phylogenetic tree to illustrate the evolution of Tektin-4 in bilaterian species. This phylogenetic analysis includes the Tektin-4 sequences identified in bilaterian species. The 23 spiralians include six mollusks (Ac, Bg, Ob, Lg, Cg, Pf), 12 platyhelminthes (Cs, Ov, Sch, Scm, Scj, Eg, Emu, Taa, Tas, Hmi, Sme, and Mli), three annelids (Pd, Ct, Hr), and one brachiopod (La). The ecdysozoans include many insects, two chelicerates (Pt, Smi), one myriapod (Sma), and one priapulid (Pc). The invertebrate deuterostomes are represented by one echinoderm (Sp), one hemichordate (Sk), one cephalochordate (Bf), and one urochordate (Ci). The vertebrate deuterostomes are represented by three teleost fish (Dr, Tr, and Ch), one holostei fish (Lo), one chondrichthyes (Cm), one sarcopterygian fish (Lc), three amphibian (Xl, Amb, Nvi), one reptile (Cpb) two avians (Ap, Gg), and three mammal species (Oa, Mm, Hs). In addition, Tektin-3/5 sequences from *B. floridae, S. kowalevskii, S. purpuratus, D. melanogaster* and *T. castaneum* were included as an outgroup. The tree was rooted with *N. vectensis* Tektin-1/4/3/5A. Both Bayesian and Maximum Likelihood analyses were performed using Mr. Bayes and RAxML, respectively. The Bayesian tree topology is shown. Node support is shown for non-terminal nodes. Posterior probability values from Mr. Bayes and bootstrap values from RAxML are shown above or below each node, respectively. Diamonds indicate support less than 80%. “X” under a node indicates that this node was not recovered in the RAxML maximum likelihood tree. The large colored boxes group Tektin-4 sequences of the three major bilaterian branches, the spiralians (green), the ecdysozoans (blue), and the deuterostomes (orange). The smaller colored boxes within ecdysozoans and spiralians highlight two gene duplications of *tektin-4* at the base of insect lepidopterans (purple), and one duplication in the Platyhelminthes lineage including the planarian Sm and the parasitic trematodes and cestodes, but excluding the flatworm Mli (dark green), respectively. Note that only two platyhelminthes species retained both Tektin-4a and -4b (Sme and Cs), while others lost one of the duplicates (Eg, Emu, Ov, Taa, Tas, Hmi, Sch, Scm, Scj). Note that *tektin-4* exists as a single copy gene in most extant species with only one additional duplicated *Tektin-4* gene observed in the spiralian leech (Hr). Species abbreviations and accession numbers for each sequence are provided in Additional file 5

Most likely the ancestral bilaterian *tektin-4* gene arose via a second duplication of the ancestral metazoan *tektin-1/4/3/5* gene. After an initial duplication gave rise to *tektin-1* and a *proto-tektin-4/3/5*, a second duplication of the latter gave rise to the two ancestral bilaterian *tektin-4* and *tektin-3/5* genes (Figs. 3 and 9). Intriguingly, the earliest branching bilaterians, the Xenacoelamorpha, may have retained a true proto-tektin-4/3/5 gene that strongly resembles tektin-4 of other bilaterians. Among the 88 bilaterian species surveyed, only the spiralians *I. linei* and *D. japonicum* and the ecdysozoan tardigrades, crustacean *D. pulex* and some chelicerates lack *tektin-4*, having apparently lost their *tektin-4* gene independently (Fig. 7, Table 1, Additional files 1, 6, 7 and 8). The only *tektin* gene present in surveyed nematodes and onychophorans is likely to be a *tektin-4* gene as indicated by sequence considerations including reciprocal BLASTP searches (data not shown) and an ecdysozoan specific phylogenetic analysis (Additional file 8), but phylogenetic analyses including taxa from other lineages did not result in reliable clustering with other *tektin-4 s*, and may alternatively suggest that the nematode *tektin* gene represents instead a highly derived *tektin-3/5*. Only four instances for retained duplications of the *tektin-4* gene are observed in bilaterians. The leech *H. robusta* has two paralogs of *tektin-4*, indicating an independent duplication in this annelid lineage (Additional file 7). Interestingly, members of the insect order Lepidoptera have as many as three *tektin-4* paralogs that cluster together with high support, indicating two duplication events in the lepidopteran lineage at the base of this taxon (Fig. 7). Similar to the findings with *tektin-2*, our analysis also suggests a *tektin-4* duplication in the spiralian platyhelminthes lineage after the split from the more basally branching *M. lignano,* but before the split between the Adiaphanida and Neodermata (Additional file 7). Interestingly, only two platyhelminthes species *(S. mediterranea* and *C. sinensis*) retained both *tektin-4a* and *− 4b* while the eight other surveyed platyhelminthes species lost one of the two duplicates. Members of the cestodes and the trematode *Opisthorchis viverrini* lost *tektin-4b* while other members of the trematodes lost *tektin-4a*. Similar to our findings for *tektin-1* and *tektin-2*, *tektin-4 s* are generally highly conserved in deuterostomes, most spiralians, and the ecdysozoan priapulid *P. caudatus* as indicated by short branch lengths and high sequence identity (~ 50–65%). In contrast, arthropod *tektin-4 s* exhibit much longer branch lengths and lower sequence identity when compared with spiralians and deuterostomes (mid-30%) (Additional file 3).

### VII. Origin and evolution of the bilaterian tektin-3/5 gene

Bilaterian *tektin-3/5* forms a sister group with *tektin-4* with high support, indicating that the *tektin-3/5* and *tektin-4* gene arose from the most recent duplication before the last common protostome/deuterostome ancestor (Figs. 3 and 9). Interestingly, *tektin-3/5* appears to be the most widely conserved bilaterian *tektin* gene in regard to sequence conservation and gene loss, with only *D. pulex* definitely lacking an ortholog, while the single extant nematode *tektin* may possibly be a *tektin-3/5* ortholog (see above). In contrast to the sequence divergence observed for *tektin-1*, *− 2* and *-4* in ecdysozoans, *tektin-3/5 s* appear to be similarly well conserved with similar branch lengths and protein sequence identity (mid-40% to mid-50%) within all three major bilaterian branches, the ecdysozoans, spiralians and deuterostomes (Additional file 3).

**Fig. 8:**
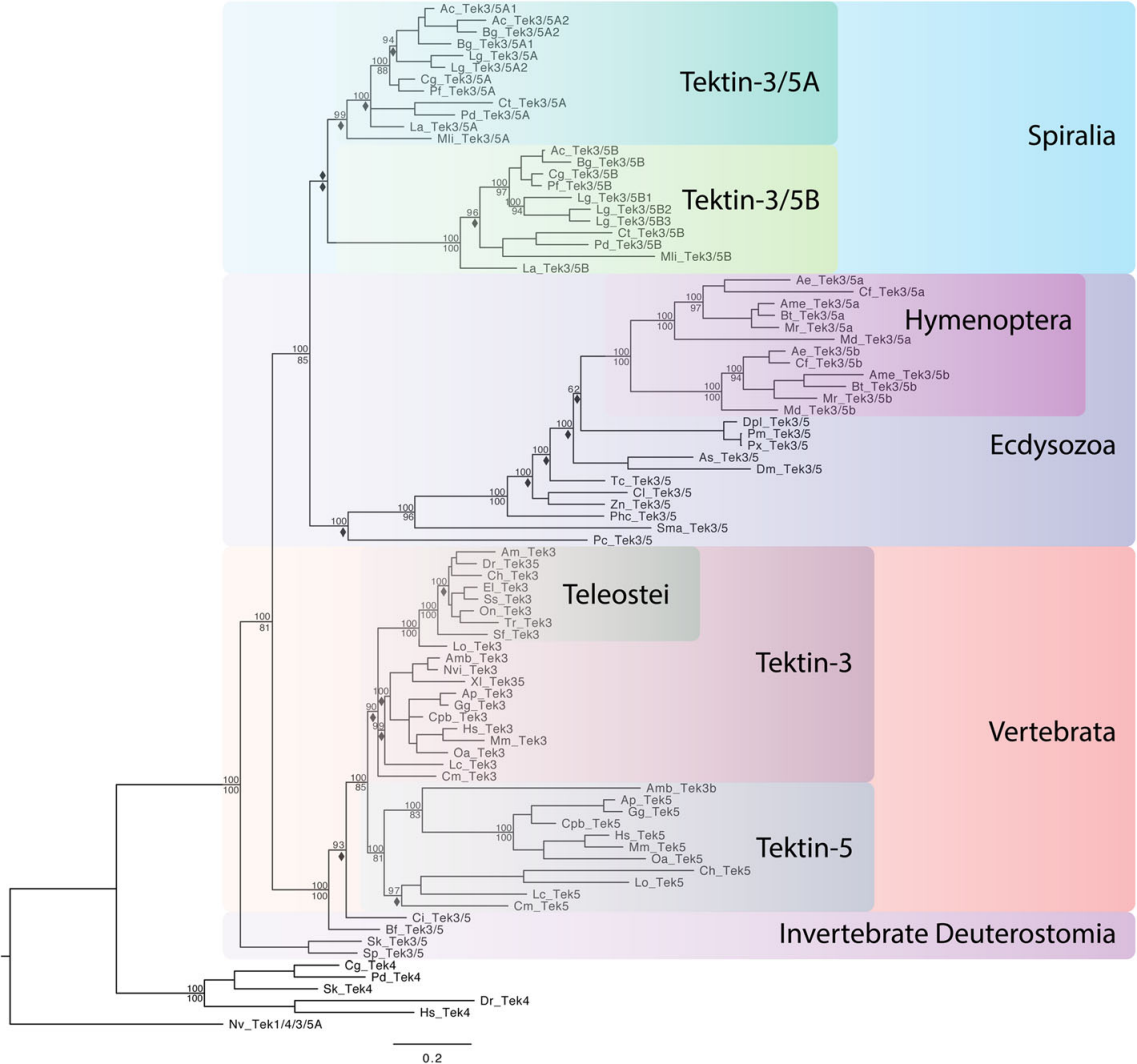
Phylogenetic tree to illustrate the evolution of Tektin-3/5 in bilaterian species. This phylogenetic analysis includes the Tektin-3/5 sequences identified in bilaterian species. The nine spiralians include five mollusks (Ac, Bg, Lg, Cg, Pf), one platyhelminthes (Mli), two annelids (Pd, Ct), and one brachiopod (La). The 17 ecdysozoans include many insects, one myriapod (Sma), and one priapulid (Pc). The invertebrate deuterostomes are represented by one echinoderm (Sp), one hemichordate (Sk), one cephalochordate (Bf), and one urochordate (Ci). The vertebrate deuterostomes are represented by eight teleost fish (Am, El, Sf, Ss, On, Dr., Tr, and Ch), one holostei fish (Lo), one chondrichthyes (Cm), one sarcopterygian fish (Lc), three amphibians (Xl, Amb, Nvi), one reptile (Cpb), two avians (Ap, Gg), and three mammal species (Oa, Mm, Hs). In addition, Tektin-4 sequences from *C. gigas, P. dumerilii, S. kowalevskii, D. rerio* and *H. sapiens* were included as an outgroup. The tree was rooted with *N. vectensis* Tektin-1/4/3/5A. Both Bayesian and Maximum Likelihood analyses were performed using Mr. Bayes and RAxML, respectively. The Bayesian tree topology is shown. Node support is shown for non-terminal nodes. Posterior probability values from Mr. Bayes and bootstrap values from RAxML are shown above or below each node, respectively. Diamonds indicate support less than 80%. “X” under a node indicates that this node was not recovered in the RAxML maximum likelihood tree. The large colored boxes group Tektin-3/5 sequences of major bilaterian branches, the spiralians (light blue), the ecdysozoans (purple), the invertebrate deuterostomes (pink), and the deuterostome vertebrates (orange). The smaller colored boxes within spiralians, ecdysozoans and vertebrates highlight three independent gene duplications of the *tektin-3/5* gene at the base of spiralians giving rise to Tektin-3/5A (dark green) and tektin-3/5B (light green), at the base of insect hymenopterans giving rise to Tektin-3/5a and − 3/5b (dark pink), and at the base of vertebrates giving rise to Tektin-3 (dark purple) and Tektin-5 (light grey), respectively. Note that most teleost fish species (dark grey), and two of the three amphibian species (Nvi and Xl) retained Tektin-3 but lost Tektin-5. Note that the *tektin-3/5* gene exists as a single copy gene in most ecdysozoans and the four invertebrate deuterostomes (Sp, Sk, Bf, Ci). Species abbreviations and accession numbers for each sequence are provided in Additional file 5

In regard to gene gain, the *tektin-3/5* gene is the most commonly duplicated *tektin* gene throughout bilaterian evolution, as it is the only bilaterian *tektin* that was duplicated at least once in each of the three major bilaterian lineages, with at least 11 additional independent duplications in several spiralian lineages (Fig. 8, Table 1, Additional files 1, 6, 7). Our analysis indicates that the *tektin-3/5* gene duplicated early in the spiralian lineage. To confirm this we performed a comprehensive analysis of spiralian *tektins* including members of the Gnathifera that are regarded as the most basal spiralian lineage [48, 49]. The micrognathozoan *Limnognathia maerski* has both a *tektin-3/5A* and *tektin-3/5B*, but two species of rotifers have only *tektin-3/5A* with *tektin-3/5B* apparently having been lost. Therefore, the last common ancestor of the spiralians already possessed two *tektin-3/5 s*: *tektin-3/5A* and *tektin-3/5B* (Fig. 8 and Additional file 7). Most spiralian species we surveyed retain at least one ortholog of both *tektin-3/5A* and *− 3/5B* with the exception of the aforementioned rotifers, the cephalopod *O. bimaculoides* that has three paralogs of *tektin-3/5A* but not *B,* and the platyhelminthes class Cestoda which have one ortholog of *tektin-3/5A*, but apparently lost the *tektin-3/5B* gene. *Dicyema japonicum* is the only spiralian identified that lacks any *tektin-3/5* homologs. Even the highly derived orthonectid *I. linei* retains single orthologs of both *tektin-3/5A* and *− 3/5B*. Several lineages have also undergone additional independent duplications of *tektin-3/5A* or *− 3/5B*. The leech *H. robusta*, the cephalopod *O. bimaculoides* and the flatworm *S. mediterranea* each have three *tektin-3/5A*, while the flatworm *P. vittatus* and the gastropods *L. gigantea, A. californica* and *B. galabrata* have two *tektin-3/5A* paralogs. The gastropod, *L. gigantea,* and the flatworm *P. vittatus* are unique among spiralians in having undergone duplications of *tektin-3/5B* gene, retaining three and two paralogs, respectively. The micrognathozoan *L. maerski*, the polychaetes *P. dumerilii* and *C. teleta*, the bivalves *C. gigas* and *P. fucata*, members of the Platyhelminthes class Trematoda, the brachiopod *L. anatina* and the orthonectide *I. linei* retain the ancestral spiralian *tektin-3/5A* and *tektin-3/5B* complement.

While invertebrate deuterostomes including the echinoderm *S. purpuratus*, the hemichordate *S. kowalevskii*, and the chordates *B. floridae* and *C. intestinalis,* all retain a single *tektin-3/5* gene, vertebrates underwent a duplication early in their lineage, likely as a result of one of the two ancient whole genome duplication, which gave rise to the vertebrate *tektin-3* and *tektin-5* genes (Fig. 8, Table 1, Additional files 1 and 6). The earliest diverging vertebrate for which we were able to find data, the ghost shark *C. milli*, retains both a *tektin-3* and *tektin-5*, indicating these two *tektin* genes were present in the last common vertebrate ancestor. While all vertebrates we surveyed retain a *tektin-3* ortholog, we found that several species lacked *tektin-5*. Although the holostei fish *L. oculatus* retains both *tektin-3* and *tektin-5*, of the eight species of teleost fish that we surveyed, seven retained *tektin-3* but lacked *tektin-5*, while only one, *Clupea harengus*, retained both *tektin-3* and *tektin-5*. In addition, the amphibians *X. laevis* and *N. viridescens* lack *tektin-5* while retaining *tektin-3*. For a third amphibian species, the axolotl *A. mexicanum,* we identified a definite *tektin-3* ortholog, but a second, partial sequence is of dubious identity. BLAST searches indicate it is a *tektin-3* paralog, suggesting a duplication, but our phylogenetic analysis indicates it might be a *tektin-5*. In either case, the absence of *tektin-5* in most teleost fish and at least some amphibians indicates independent losses. All other vertebrates we surveyed, including the coelacanth *L. chalumnae* and the mammal *H. sapiens*, retain orthologs of both *tektin-3* and *tektin-5.*

Intriguingly, *tektin-3/5* is the most highly conserved *tektin* in ecdysozoans, with branch lengths comparable to spiralian and deuterostome *tektin-3/5 s* (Fig. 8, Table 1, Additional files 1, 6, 7 and 8) and generally higher sequence similarity compared to other Tektin proteins (Additional file 3). Our analysis indicates that the last common ecdysozoan ancestor had a single *tektin-3/5*. While *tektin-3/5* is definitely lost in *D. pulex* and possibly lost in nematodes, all other ecdysozoans that we surveyed have a single *tektin-3/5* ortholog with the exception of the six hymenopteran insect species which each have two *tektin-3/5* paralogs, named *tektin-3/5a* and *− 3/5b* which each cluster together with high support, indicating a gene duplication event at the base of the hymenopteran lineage.

## Discussion

### The emergence of tektin genes in eukaryotic and metazoan evolution

**Fig. 9:**
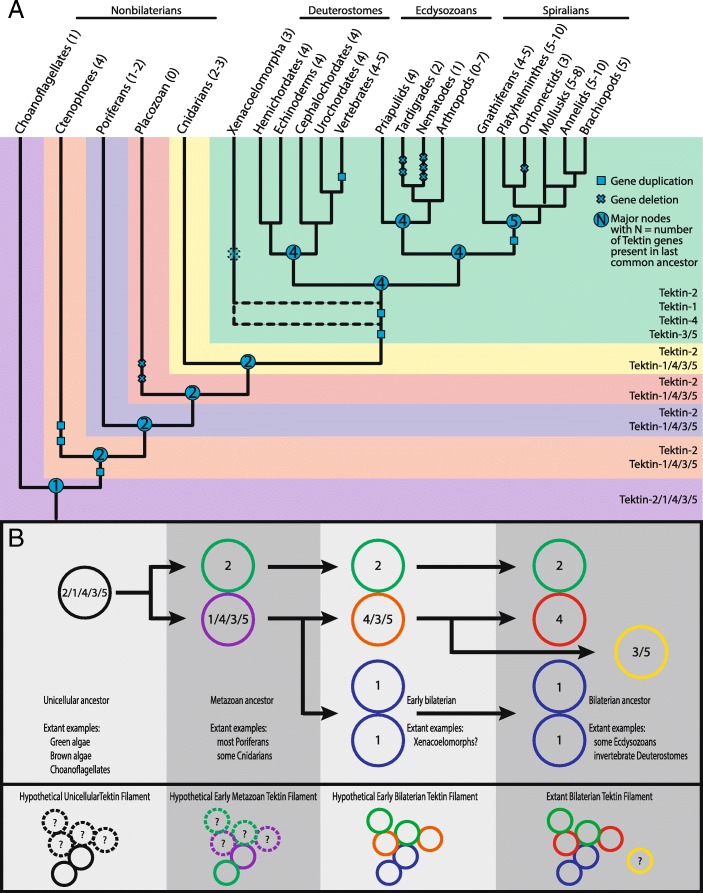
Overview of metazoan *tektin* gene family evolution. **a** Evidence-based parsimonious hypothesis of the evolution of the *tektin* gene family in metazoans. Results from this study are displayed within a mostly accepted phylogeny of metazoan taxa. The branching pattern of nonbilaterian taxa especially the ctenophores are still debated. However, the proposed scenario is consistent with either ctenophores or poriferans as the earliest branching metazoan taxon. The number of hypothesized ancestral *tektin* genes is given at each major node (blue circles) with a suggested nomenclature that reflects the evolutionary origin on the Tektin classes is given at the right margin. Note that according to our analysis the four Tektin classes, Tektin-1, − 2, − 4, and 3/5 exist only in bilaterians, while the extant bilaterian and nonbilaterian *tektin* gene complements originated from two ancestral metazoan tektins, *tektin-2* and *tektin-1/4/3/5*. Major duplications (blue squares) and losses (blue crosses) of *tektin* genes that affect every species within a phylum are shown. Metazoan phyla and in parenthesis the range of tektin genes found within species of each phylum are shown in the top row. Number of *tektin* genes range from complete absence in placozoans (0) by gene loss, to ten tektin genes in the annelid leech (10) by multiple gene duplications. Note that three spiralian taxa, the platyhelminthes (5–10), the annelids (5–10), and the mollusks (5–8) contain each species with the ancestral spiralian *tektin* gene complement of five, and species that have expanded their complement up to ten *tektin* genes by independent gene duplications. Uncertainty of the placement of the Xenacoelomorpha either before or after the duplication of *tektin-4/3/5* leading to *tektin-4* and *tektin-3/5* is indicated by dotted lines. **b** Hypothesized scenario for the evolution of the Tektin filament. Pioneering biochemical and structural studies on tektins in motile cilia of sea urchins by Linck and colleagues have provided evidence for axonemal Tektin filaments constructed by multiples of two heterodimers built with Tektin-2 (green circle) and Tektin-4 (red circle), and one homodimer built with Tektin-1 (blue circle) protein units (compare to Fig. 1c). This phylogenetic study provided evidence that these three Tektins existed already in a bilaterian ancestor including a fourth Tektin-3/5 (yellow circle) with unknown filamentous Tektin functions (shown in right column), and enables predictions about the composition for earlier stages in Tektin filament evolution with the ancestral *tektin* gene complements comprising of one (*tektin-2/1/4/3/5*, black circle), two (*tektin-2*, green circle; *tektin-1/4/3/5*, purple circle), and three *tektin* genes in the unicellular, metazoan, and early bilaterian ancestor, respectively (shown in three left columns). Upper row shows which predicted Tektin protein makes up the filamentous Tektin units at discreet steps in metazoan evolution, and lists extant organisms in which this prediction could be tested. Lower row shows diagrams of the predicted composition of the hypothetical Tektin filament at discreet steps in evolution

Our comprehensive sampling and phylogenetic analyses of the *tektin* gene complement in eukaryotic genomes enabled the inference of discreet steps in the emergence of the *tektin* genes during evolution (Fig. 9a). (1) The presence of single copy *tektin* genes in only three distantly related eukaryotic branches favors some form of horizontal gene transfer. (2) All extant metazoan *tektin* genes arose via duplication from a single ancestral *tektin* gene present in the last common ancestor of metazoans and choanoflagellates. Choanoflagellates retain this single ancestral *tektin* gene. (3) Duplication of this ancestral *tektin* gene coincides with the transition from unicellular to multicellular organisms at the base of the metazoan lineage generating to two ancestral metazoan *tektin* genes, *tektin-2* and *tektin-1/4/3/5.* Most extant sponges retain these two ancestral *tektin* genes. (4) Repeated duplications of the ancestral *tektin-1/4/3/5* gene led to independent expansions of the *tektin* gene complements in ctenophores, some cnidarians and the bilaterians. (5) The earliest branching bilaterians may have possessed three *tektin* genes, while the ancestor for all other bilaterians (Nephrozoa) possessed four *tektin* genes. *Tektin-2* which is orthologous to the *tektin-2* found in nonbilaterian metazoans, and *tektin-1, tektin-4* and *tektin-3/5* which arose via multiple duplications of *tektin-1/4/3/5* prior to the divergence of protostomes and deuterostomes. This ancestral bilaterian complement of four *tektin* genes corresponds to the four major classes of bilaterian *tektin* genes that was maintained by some and modified by other extant bilaterian species. One of the remarkable findings of this study is the well-defined expansion from the ancestral metazoan to the ancestral bilaterian and nephrozoan *tektin* complement from two to three and four *tektin* genes, respectively, that is well supported by phylogenetic inference. Equally insightful are the intriguing modifications of these ancestral *tektin* complements in various extant metazoan species by *tektin* gene loss or gain in the context of our current understanding of Tektin function.

### Previous phylogenetic analyses of the tektin gene family

Prior to this study there have been only two notable attempts to establish the evolutionary history of the *tektin* gene family proteins, both of which have serious shortcomings. The more comprehensive phylogenetic analysis of the two by Amos [20] was hampered by a lack of available sequence data from several important metazoan lineages, while the more recent one by Nevers et al. [50] used an automated approach to assign *tektins* to orthologous groups as part of an analysis to determine the evolution of hundreds of distinct cilia-related gene families. A side-by-side comparison of the results of our study and these two previous studies is given in Additional file 9. We compare common species between the studies where possible, and in a few instances compared species of the same genus, family or order.

In comparison to our study Amos [20] missed many *tektin* homologs. Only the results for mouse, frog and green algae are consistent with our study. For all other comparable species *tektin* homologs were either missing or misassigned. Amos suggested that humans possess at least six *tektins*, and her phylogenetic analysis indicated as many as ten. It should be noted that no other study has identified more than five human *tektins*. Upon closer inspection, we have confirmed that each of the additional human *tektins* found by Amos have since been deleted from the record and/or classified as pseudogenes. While Amos [20] did not explicitly recognize the *tektin-3/5* class, the placing of invertebrate sequences within the tree topology as sister group to vertebrate *tektin-3* and *tektin-5* is nevertheless consistent with our study. Amos [20] also indicates that *tektin-1* and *tektin-4* are closer related, while *tektin-3/5* is a sister group to both, whereas our study clusters *tektin-4* and *tektin-3/5* together with high support and *tektin-1* as a sister group to both. Both studies agree that *tektin-2* is the earliest diverging bilaterian *tektin*.

Comparison with Nevers et al. [50] is more complicated as their methodology attempted to assign all *tektins* as orthologs to one of the five vertebrate *tektin* classes utilizing an automated bioinformatics approach. Our analysis indicates that this approach fails to assign *tektin* orthology correctly most likely due to duplications and varied sequence divergence of *tektin* genes in distinct species and taxa during metazoan evolution. In addition, Nevers et al. [50] display their results only as *tektin* genes present or absent in each species. Our results are consistent with the Nevers et al. [50] for most unicellular organisms with the exception of the choanoflagellate *S. rosetta*. While we found only one *S. rosetta tektin* homolog, Nevers et al. [50] indicates one orthologous *tektin* gene for each of the five vertebrate *tektin* classes. Despite intensive searches within *S. rosetta* genomic and proteomic data, we have been unable to identify more than one potential *tektin* homolog, and therefore suggest that Nevers et al. [50] is in error. Among metazoans, only the vertebrate *tektin* complements showed any degree of consistency between these two studies, as would be expected given the methodology used by Nevers et al. [50]. As Nevers et al. [50] did not account for the *tektin-3/5* class or the duplications early in the bilaterian lineage, all results for invertebrates are inconsistent and incompatible between these two studies. It should be noted that both Amos [20] and Nevers et al. [50] were lacking broader sampling of spiralian data, with both including only flatworms from the highly derived *Schistosoma* genus. Thus, the comparison between these three studies indicates that (1) *tektin* phylogeny was in severe need of an update since Amos ([20], 2) that despite the growing popularity and usefulness of large scale analyses such as Nevers et al. [50], it is still imperative to perform careful, in depth studies of individual protein families in order to produce an accurate description of their evolutionary history. While this manuscript was prepared another large scale analysis of cilia related proteins was published [51] which also included Tektin proteins as part of a larger, automated phylogenetic analysis of ciliary genes. However, in regard to Tektins this analysis was largely similar to Nevers et al. [50], and suffered from many of the same shortcomings.

### Motile cilia first, tektin later?

Intriguingly, *tektin* genes are present in only three of over 18 major unicellular eukaryotic lineages that possess motile cilia. These three Tektin-possessing lineages, chlorophytes, cryptophytes, and apoikozoans (choanoflagellates and metazoans), are phylogenetically widely separated by over a dozen eukaryotic lineages with motile cilia and/or flagella [52] but without any *tektin* gene. The most parsimonious interpretation of this distribution is that the last common unicellular ancestor with cilia did not possess *tektin* genes at all, and that *tektin* genes appeared and acquired an essential function in cilia later during eukaryotic evolution., Furthermore, this scenario suggests two or more exchanges of the *tektin* gene by horizontal gene transfer between these three eukaryotic lineages*.*

### Implications for the origin and evolution of the Tektin filament

Pioneering biochemical and structural studies have revealed the composition of the Tektin filaments in motile cilia in sea urchins thought to contain an equal molarity of Tektin-1, − 2, and Tektin-4 proteins corresponding to Tektin-C, -B, and –A, respectively [21–24]. Currently, no contribution of Tektin 3/5 proteins to the Tektin filaments have been reported, nevertheless more targeted studies on the localization of Tektin-3/5 are required. Thus, these filaments are apparently constructed of multiples of two heterodimers (1xTektin-2 and 1xTektin-4) and one homodimer (2xTektin-1). These filaments are thought to provide structural support within the axoneme, and might function as ‘rulers’ to regulate the length of motile cilia [21, 22]. Although our current understanding of Tektin function in other species than sea urchin is rather limited, the delineated pattern of *tektin* gene evolution unraveled in this study predicts distinct changes in the composition of the Tektin filament in distinct metazoan lineages (Fig. 9b). As the composition of the Tektin filament of sea urchin utilizes – besides the widely conserved metazoan Tektin-2 - two quintessential bilaterian Tektins (Tektin-1 and -4) that originated from an ancestral metazoan Tektin (1/4/3/5), the composition of the filament in ancestral metazoans and in extant pre-bilaterians must be different. One testable prediction would be that Tektin filaments in unicellular eukaryotes that possess one *tektin* gene like the chlorophyte *C. reinhardtii* and choanoflagellates are comprised of homodimers, while in extant sponges that possess two *tektin* genes filaments are constructed by heterodimers (Fig. 9b).

### Do filamentous tektin genes ‘coevolve’ within bilaterians?

Given the structural composition of the Tektin filaments by Tektin-1, − 2, and − 4 units while Tektin-3/5 has to date not been shown to play a role in filament formation, it is intriguing that the three filamentous *tektin* genes share similar evolutionary trajectories in various bilaterian lineages. *Tektin-1, − 2,* and *− 4* appear to be rarely duplicated or lost. Only two hexapod lineages (lepidopterans and anoplurans), the leech *H. robusta,* the planarian *S. mediterranea*, and several species of the highly derived parasitic flatworms have duplications of *tektin-2*, while the leech *H. robusta,* some flatworms, and the lepidopterans have duplications of *tektin-4*. Only the flatworm *S. mediterranea* and the leech *H. robusta* possess duplications of *tektin-1*. All other ecdysozoans and spiralians, as well as all deuterostomes that we surveyed retain at most one extant ortholog each of *tektin-1, − 2* and *− 4*. This retention and conservation as single copy genes is especially remarkable in the vertebrate lineage where comparative genomics has firmly established two early whole genome duplication events prior to the vertebrate radiation [37, 38]. While many other gene families including the hox genes have retained many duplicated genes, all of the duplicated *tektin-1, − 2,* and *− 4* genes in the vertebrate ancestor were apparently rapidly lost, suggesting some constraints to increasing the *tektin* complement.

Another striking outcome of our analysis are the consistently long branches of *tektin-1, − 2*, and *− 4* in the ecdysozoans. Whereas spiralian and deuterostome orthologous Tektins are highly conserved at the protein sequence level, e.g. spiralian Tektins sharing over 50% identity with their deuterostome orthologs and often greater than 40% identity with other Tektin paralogs, among ecdysozoans only Tektin-3/5 regularly shares greater than 40% identity with orthologs in species from the two other major bilaterian superphyla (Additional file 3). Thus, ecdysozoan *tektin-1, tektin-2* and *tektin-4* appear to have diverged rapidly from the ancestral sequence suggesting the loss of some evolutionary constraint at the base of the ecdysozoans or arthropods. In addition, several independent losses of all but one *tektin* gene in the crustacean *D. pulex,* onychophorans and nematodes, and loss of all *tektins* in some chelicerates, may also indicate the loss of some common constraint within ecdysozoans compared to spiralians and deuterostomes ‘filamentous’ *tektins*.

Thus, ‘filamentous’ *tektin* genes appear to have coevolved within different bilaterian lineages, stayed highly conserved as single copy genes in deuterostomes and most spiralian species, but strongly diverged or were lost in ecdysozoans. It is tempting to speculate that the common constraint of these genes in deuterostomes and spiralians is due to the retention of a common interdependent function of these proteins within a Tektin filament in motile cilia and/or sperm flagella. On the other hand, the general sequence divergence in ecdysozoans as well as the frequent independent loss to tektin genes in nematodes, crustaceans and tardigrades may indicate loss or novel functions for the filamentous *tektins*. Indeed, a lack of motile cilia is regarded as a diagnostic feature of ecdysozoans [53], and nematodes and some crustaceans are well known for their aflagellar sperm morphology [54, 55].

Thus, it would be interesting to investigate how the divergence and reduction of the bilaterian *tektin* complements in ecdysozoans has affected the make-up of Tektin filaments and axonemes. Are Tektin filaments completely lost from the axonemes of all cilia? Are Tektin filaments only utilized in specialized cilia in ecdysozoans, and if yes is the filament’s composition in nematodes and the crustacean *D. pulex* now comprised of their single remaining Tektins only?

### Are ‘filamentous’ tektin genes required for motile cilia function?

While the widespread presence and conservation of filamentous *tektin* genes in metazoans argue for some essential, conserved and ancient ciliary functions, genetic and/or other functional evidence is still scarce. Most of the *tektin* studies to date have focused on their role in sperm flagella in mouse, rat and sea urchin as well as cilia formation in sea urchin. There is functional evidence in mouse and sea urchin that *tektins* have a necessary role for motility of both sperm flagella and ‘motile cilia’, respectively [25–27, 56, 57], and that *tektin* mutations and dysfunction contribute to flagellar defects in mammalian sperm [25–27] and the unicellular algae *C. reinhardtii* as well [19].

However, *tektin* genes are surprisingly absent in most eukaryotic lineages that are known to possess motile cilia including the ciliates (*Tetrahymena, Paramecium*) and the apicomplexan (*Plasmodium*), but also the placozoan *Trichoplax adhaerens*, the only metazoan species without any *tektin* genes, and demonstrate that these species do not need a highly conserved *tektin* gene for ciliary functions. This raises the interesting question as to how these species are able to produce functional motile cilia without the presence of *tektins,* while metazoans with the notable exceptions of placozoans apparently require it? One possibility to solve this conundrum could be that *tektin* genes are only required for distinct types of ‘motile’ cilia and flagella that are characterized by certain length, and/or by higher force generation. Alternatively, eukaryotes without *tektin* including ciliates, apicomplexan, and placozoans may utilize compensatory molecular mechanisms to fulfill analogous Tektin functions within their ‘motile’ cilia. Thus, these species may represent a key avenue of research into how organisms evolved new types of motile cilia without key structural proteins necessary for ciliary function in other animals.

In this context, one should also discuss metazoan lineages that lost multiple *tektins* especially the nematodes and some crustaceans. These species are known to have aflagellar sperm morphology [54, 55], suggesting that loss of *tektins* might correlate with loss or reduction of flagellar sperm functions. In this regard, it is intriguing that the presence of ‘sperm cells’ is still controversial in placozoans [58, 59].

### Ancestral and novel roles for tektin-3/5 in bilaterians?

Our phylogenetic analyses suggest that the *tektin-3/5* gene represents the latest addition to the *tektin* gene family within bilaterians, being absent from prebilaterian lineages, and originating from a duplication of the ancestral *tektin-4/3/5* gene. Intriguingly, the evolutionary trajectory of the *tektin-3/5* gene is more diverse compared to the other three bilaterian *tektin* genes, exhibiting more frequent gene duplications within the various bilaterian lineages. Thus, despite the sequence constrain, tektin-3/5 appears less evolutionary constrained in regard to gene duplication compared to the other *tektin* genes. Whether this can be attributed to novel functions demands more targeted studies. Data in sea urchins identifies only Tektin-1, − 2 and − 4 proteins as components of the Tektin filament, but this does not exclude Tektin-3/5 from any filamentous function, although localization patterns for Tektin-3/− 5 protein in the periphery of the axoneme in vertebrates [60] suggests diverging roles. While mutations of the *tektin-3/5* paralog *tektin-*3 in mammals have been associated with structural defects in sperm flagella, it did not negatively impact fertility [26]. Additional studies are certainly needed to elucidate its actual role in sperm flagella and/or motile cilia.

Duplications of the bilaterian *tektin-3/5* gene are most prominent in spiralian lineages. Especially intriguing is a duplication of *tektin-3/5* early in the evolution of spiralians giving rise to a *tektin-3/5A* and *− 3/5B* gene that have been retained in most annelids, mollusks, platyhelminthes, and brachiopods included in our study. Thus, the existence of these two distinct *tektin-3/5* genes may represent an intriguing and useful synapomorphy for the spiralian clade. Consistently, this study identified clear orthologs for both *tektin-3/5A* and *tektin-3/5B* genes in the orthonectid *I. linei*, a species within a taxon that a recent genomic study identified as a highly derived spiralian [61].

Our study also found frequent additional independent duplications of the *tektin-3/5* gene in several spiralian species including the leech *H. robusta,* the planarian *S. mediterranea* and cephalopod *O. bimaculoides* as well as in the three mollusk gastropod species *L. gigantea*, *A. californica* and *B. galabrata.* Thus, expansions of the *tektin-3/5* gene families have occurred apparently independently in both direct developing species (without apparent larval stages), as well as in indirect developing species (with one or more larval stages), respectively. It will be interesting to see where and how these additional *tektin-3/5* genes might be utilized. Gastropod species may use species-specific expansions of *tektin-3/5* genes for the various ciliary structures and functions of their larvae while direct developing species like planarians and leech may utilize them in specialized ciliary based sensory structures to facilitate their similar aquatic life style.

In contrast to the frequent duplications in spiralians, the *tektin-3/5* gene was retained as a single copy gene in most ecdysozoan lineages, exhibiting less divergence from the ancestral bilaterian *tektin-3/5* sequence than the three ecdysozoan ‘filamentous’ *tektin* genes (see above). As far as we know no study addressed *tektin-3/5* function in any ecdysozoan species, though it promises to yield insights into potential ancestral functions. The observed duplication at the base of the insect hymenopteran clade comprises an interesting synapomorphy among bee, wasp, and ant species.

Our study indicates that the *tektin-3/5* gene was retained as a single copy gene in invertebrate deuterostomes including the ambulacrarian echinoderm and hemichordate species, as well as the chordates *B. floridae* and *C. intestinalis*, and remained strongly conserved in sequence. Currently unknown, studies to localize and determine the function of Tektin-3/5 protein would be especially informative in these species. Our analyses indicate also that all vertebrate *tektin-3* and *-5* genes are the result of a duplication of the ancestral bilaterian *tektin-3/5* gene early in the vertebrate lineage. Thus, these genes may represent the sole surviving duplicated *tektin* genes retained from the two whole genome duplications that took place early in vertebrate evolution [37, 38]. Although current classification defines *tektin-3* and *tektin-5* as different *tektin* classes, these are clearly vertebrate specific, and we suggest designating them as members of one bilaterian *tektin-3/5* class to reflect their evolutionary history correctly. In this context it is also interesting that a knockout study of *tektin-3* in mice observed defects in sperm flagella but nevertheless retained normal fertility, while *tektin-4* knockouts had impaired fertility also [25, 26]. As studies have not yet determined the role of the *tektin-5* gene in mice, it could be that the closer related *tektin-3* and *tektin-5* retained some functional redundancy in vertebrates, requiring both to be lost before fertility is impaired.

### Several species retained ancestral tektin complements

We infer from our analysis that the last common ancestor of the protostomes and deuterostomes possessed single copy genes for four ancestral bilaterian *tektin* genes corresponding to four instead of five currently defined *tektin* classes: *tektin-1, tektin-2, tektin-4* and *tektin-3/5*, respectively. All invertebrate deuterostome species we surveyed retain this ancestral state, as do ecdysozoan priapulids and several arthropod hexapod classes including Diptera, Coleoptera, Isoptera, and some members of Hemiptera. Importantly, due to the duplication of *tektin-3/5* early in the spiralian and vertebrate lineages, no spiralians or vertebrates retain the ancestral bilaterian state. Therefore we suggest that studies to localize and determine the function of *tektin* genes would be especially informative in these species. Comparative studies of nonbilaterians with bilaterian species that have retained the ancestral *tektin* complement like the insects, *D. melanogaster* and *T. castaneum* among ecdysozoans, and especially the invertebrate deuterostomes could provide clues to ancestral *tektin* functions and offer insights into how their diversification contributed to evolutionary history and diversification of sperm and motile cilia function.

A similar argument can be made to determine ancestral functions of *tektins* in spiralians and vertebrates. Our study supports the view that the last common ancestor of the spiralia and the last common ancestor of the vertebrates each had five *tektin* genes due to independent duplications of the *tektin-3/5* gene. Among vertebrates this ancestral state is retained in all lineages except for the teleost fish and amphibians that appear to have undergone independent losses of the *tektin-5* gene. Among spiralians the gnathiferan *L. maerski,* the gastrotrich *L. squamata*, the nemertean *L. longissimus,* the annelids *P. dumerilii* and *C. teleta*, the bivalve mollusks *C. gigas* and *P. fucata*, the flatworm *M. lignano*, and the brachiopod *L. anatina* retain this ancestral spiralian state and would be prime candidates for functional studies.

We identified no members of the Xenacoelomorpha that retained four ‘bilaterian’ *tektins.* As a recent study has indicated that this phylum is the most basal bilaterian phyla and sister to the Nephrozoa [62], this could indicate that the duplication of the hypothetical ancestral *tektin-4/3/5* that gave rise to *tektin-4* and *tektin-3/5* occurred after the split of the Nephrozoa from the Xenacoelomorpha. However, in our analysis the Xenacoelomorpha retained unambiguous orthologs of *tektin-1* and *tektin-2* and a third ortholog that clustered unambiguously with the *tektin-4 s* of other species. It is possible that this *tektin-4* clustering is an artifact created by the generally longer branches and greater sequence divergence of *tektin-3/5*, while in actuality the Xenacoelomorpha’s *tektin-4* represents a *tektin-4/3/5.* Given the current evidence, it may be equally likely that the duplication occurred prior to the split of Xenacoelomorpha and Nephrozoa and that the Xenacoelomorpha *tektin-3/5* was later lost. However, if the former is correct Xenacoelomorpha may truly represent and have retained a ‘transitional’ ancestral bilaterian *tektin* complement of three.

### Species with notable expansions of the tektin gene complement

Our analysis identified several metazoan taxa with remarkable independent expansions of the ancestral *tektin* complement through duplications, including the ctenophore and bilaterian clade (two to four *tektins*), the insect lepidopterans (four to seven *tektins*), mollusk gastropods (five to six or eight *tektins*), as well as the leech *H. robusta* and the planarian *S. mediterranea* (five to ten *tektins*). These duplications may have played a role in the evolution and specialization of new cilia types or functions in these lineages. For example, ctenophores commonly named ‘comb jellies’ possess the longest known motile cilia that form their characteristic beating ‘combs’. As Tektin proteins have been shown to play a role in cilia stability and motility, extra *tektin* genes may have contributed to evolve these extraordinarily long cilia. In the case of gastropods and planarians, extra *tektins* may have played a role to facilitate their unique mode of locomotion that relies upon ciliated epithelia to glide along surfaces [18, 63, 64] and therefore independent expansion of *tektin* genes may have also played a role in making the evolution of this mode of locomotion possible. This argument is especially compelling for planarians as the closely related parasitic flatworms the cestodes and trematodes have lost some of the duplicated *tektin* genes, suggesting that this loss may reflect that *tektins* are no longer needed as these parasites no longer rely on a ciliated epithelium for locomotion. The expansion in lepidopterans is remarkable as they are members of the Ecdysozoa, a clade defined by its lack of motile cilia, and therefore the expansion to seven *tektins* must invoke perhaps novel functions. Studies in mouse and rat have indicated that *tektins* are not expressed in primary or sensory cilia in mammals [65–67]. However, the retention of a complete but divergent bilaterian *tektin* complement in ecdysozoans, and especially the expansion from four to seven *tektin* genes in lepidopterans might hint at a common yet ancient role of *tektins* for lengthy cilia in this clade perhaps in general and/or specialized sensory organs, respectively.

## Conclusions and outlook

Utilizing broader sampling of previously underrepresented taxa our study provides an updated framework that tracks *tektin* gene family evolution by gene gain and gene loss. While the unicellular holozoan ancestor possessed a single copy *tektin* gene, the *tektin* gene complement expanded to two, three, and four *tektin* genes in the metazoan, bilaterian, and nephrozoan ancestor, respectively. Our analysis suggests that classification of bilaterian *tektins* into four classes, *tektin-1, − 2, − 4,* and *− 3/5*, would be consistent with their evolutionary history, and identifies *tektin-2* as the earliest, and *tektin-3/5* as the latest emerging *tektin* genes. While *tektin-1, tektin-2* and *tektin-4* remained single copy genes in many bilaterians, additional gene duplications occurred more frequently in the *tektin-3/5* lineage. Specifically, our study identified expansions, reductions, and sequence divergence of the *tektin* gene complement for over 100 extant species. Intriguingly, our study identified complete loss (in placozoans), extensive expansions (in planarians, in leech, and in lepidopterans), as well as ancestral conservation (in deuterostome invertebrates) of the *tektin* gene complement, suggesting several candidate species for future studies. More studies that investigate the entire *tektin* gene complement within informative species are needed to elucidate the various roles of the *tektin* gene family members for various ciliary functions within diverse metazoans. Investigations should determine the expression of *tektin* transcripts, localization of Tektin proteins, and functional studies in informative species to gain insights into ‘filamentous’ and/or other *tektin* functions. No comprehensive studies of *tektin* expression and/or function have yet been done in any protostome species. Especially spiralians, whose various larval stages utilize arrays of multi-ciliated cells called ‘ciliary bands,’ offer a fertile ground to explore the role of *tektins* to generate different types of cilia and ciliary functions, as well as to how *tektin* gene duplications and loss may have contributed to cilia and flagellar diversity.

## Methods

### Species selection and sequence retrieval

Species were selected to represent all major metazoan phyla as well as non-metazoan lineages. Attempts were made to obtain sequences from representatives of all metazoan phyla as well as taxa comprised of unicellular organisms known to have motile cilia and/or flagella. Tektin proteins were identified by reciprocal BLAST analysis using annotated *H. sapiens* Tektin protein sequences as queries against protein, transcriptomic and genomic sequence databases. More sensitive DELTA- and PSI-BLAST searches were used to confirm lack of Tektins in species and lineages for which BLASTP and T-BLASTN searches failed to identify any Tektin sequences. *P. dumerilii* Tektin sequences were obtained from transcriptomic data [68]. A total of 439 Tektin protein sequences were obtained from 111 species representing 27 phyla including 24 metazoan phyla and *Cryptophyta, Chlorophyta* and *Choanoflagellata* among non-metazoans. For a comprehensive list of species used, the sources, as well as the sequence data see Additional files 2 and 5.

### Alignment and phylogenetic analysis

Multiple alignments were performed with MAFFT [69] using the MAFFT iterative approach (MAFFT L-INS-i) [70]. Alignments were visualized and divergent ends were trimmed using Aliview [71]. Any positions with 70% or more gaps were removed (Additional file 4). Bayesian phylogenetic analysis was performed using Mr. Bayes ver 3.2.6 with the mixed amino acid substitution model with a proportion of invariant sites and gamma distribution (invgamma) [41]. Analyses were run for 2,000,000 generations with a 25% burn in. Maximum Likelihood analyses were performed using RAxML ver 8 [40]. Because RAxML does not offer a mixed amino acid substitution model, the Tektin alignment was submitted to the Prottest-3 server [72] for selection of the best model. LG substitution model with proportion of invariant sites and gamma distribution was selected (LG + I + G). Maximum Likelihood analyses were run for 1000 bootstraps and the best scoring tree was selected. Trees were visualized in FigTree (http://tree.bio.ed.ac.uk/software/figtree/). Tree figures were modified for publication in Adobe Illustrator. Very short sequences, sequences producing very large branches and unstable sequences not consistently clustering with any particular group in preliminary analyses were left out of final analyses.

## Additional files

Additional file 1: Comprehensive table of the *tektin* gene complement found in species included in this study (related to Table 1). The table shows all species examined in this study and their *tektin* gene complement with the exception of some teleost fish removed for redundancy. Species are listed according to four groups: (1) nonbilaterians, (2) spiralians, (3) ecdysozoans, and (4) deuterostomes. The *tektin* gene complement for each species is presented by total number of *tektin* genes within their genome in the right column, as well as the number of homologs found for each of the four bilaterian classes: Tektin-2, Tektin-1, Tektin-4, and Tektin-3/5 (in spiralians: Tektin-3/5A and 3/5B, in vertebrates: Tektin-3 and -5). The *tektin* gene complements for distinct inferred Last Common Ancestors (LCAs) are also shown. Gray boxes indicate metazoans that have maintained the ancestral *tektin* gene complement inferred for their clade (nonbilaterians, spiralians, ecdysozoans, or deuterostomes). Blue boxes indicate metazoans that have experienced duplications of *tektin-3/5*, but maintain single copies of *tektin-1, − 2* and *− 4*. Pink boxes indicate the vertebrates that have lost *tektin-5.* Note that the four Tektin classes are bilaterian specific originating by ancient gene duplications from two nonbilaterian and one unicellular *tektin* gene(s). * indicates partial sequence left out of final analysis. ** indicates highly divergent long branch sequences after initial analyses and left out of final analysis. Yellow circles indicate key clades as follows. Unicellular Eukaryotes and Nonbilaterian Metazoans table: E = Eukaryota, O = Opisthokonta, H = Holozoa, A = Apoikozoa, M = Metazoa. Spiralian table: S = Spiralia, G = Gnathifera, R = Rouphozoa, M = Mesozoa, L = Lophotrochozoa. Ecdysozoan table: E = Ecdysozoa, A = Arthropoda. Deuterostome table: D = Deuterostomia, C = Chordata. Tree structures are based on – Eukaryotes: Burki 2014, Janouskevec et al. 2017, Torruella et al. 2015, and Budd and Jensen 2017 [73–76]; Spiralia: Laumer et al. 2015 and Lu et al. 2017 [49, 77]; Ecdysozoa: Borner 2014 [78]. * = for alternative scenario of the bilaterian ancestor see Discussion. (PDF 223 kb)

Additional file 2: Fasta file of Tektin protein sequences examined in this study. (FASTA 441 kb)

Additional file 3: Conservation and Divergence of Tektin proteins in bilaterians. Comparison of Tektin sequence conservation among selected species from spiralians (Mli, Cg, La, Pd), ecdysozoans (Tc, Bt, Dm, Pc, Sma), and deuterostomes (Hs, Gg, Bf, Sp, Sk). Percent identity shared at the amino acid level is given for each Tektin. Boxes are shaded according to degree of conservation with 30% identity or below being white, and 70% and above being the darkest blue. While spiralian and deuterostome exhibit high levels of sequence conservation at the amino acid level between Tektins of each class, ecdysozoans tend to have much lower sequence identity both when compared to spiralians and deuterostomes as well as when compared to other ecdysozoans. Exceptions are ecdysozoan Tektin-3/5 s that are generally higher conserved than other ecdysozoan Tektins belonging to the Tektin-1, − 2, and − 4 class. (PDF 628 kb)

Additional file 4: Amino acid alignment of Tektin proteins. Nexus file containing multiple sequence alignment of Tektin proteins. Sequences were aligned with MAFFT using the MAFFT L-INS-i approach. Alignment was trimmed in Aliview (see Methods). (NEXUS 139 kb)

Additional file 5: List of species, source of sequences, and accession numbers of Tektins. List of all species examined in this study with names and accession numbers and/or other sequence identifiers for each Tektin identified. The source is given for the origin of each sequence obtained. * indicates partial sequence left out of final analysis. ** indicates highly divergent long branch sequences in initial analyses and left out of final analysis. Most sequences come from either NCBI or ENSEMBL. Other sequence sources include parasite.wormbase.org [79, 80], compagen.org [81], smedgd.stowers.org [82], neurobase.rc.ufl.edu, marinegenomics.oist.jp [83, 84], sandberg.cmb.ki.se [85], Mnemiopsis Genome Project Portal [86, 87], tardigrades.org, ambystoma.org [88, 89], or transcriptomic data provided by Dr. Andreas Hejnol used in Cannon et al*,* 2016 [62]. (DOCX 44 kb)

Additional file 6: Comprehensive phylogenetic tree of the *tektin* gene family (related to Figs. 3 and 4). This comprehensive phylogenetic analysis includes species representing all major metazoan lineages, choanoflagellates and algae. Both Bayesian and Maximum Likelihood analyses were performed using Mr. Bayes and RAxML, respectively. Bayesian tree is shown. Node support is shown for non-terminal nodes. Posterior probability values from Mr. Bayes are shown above each node and bootstrap values from RAxML are shown below each node. Diamonds indicate support less than 80%. An “X” under a node indicates this node was not recovered in the RAxML maximum likelihood tree. Tree was rooted with the brown algae *G. theta* (Chlorophyta). The topology of the tree indicates that the last common ancestor of choanoflagellates and metazoans had a single *tektin* gene. Subsequent gene duplications gave rise to two and four *tektin* genes in the metazoan and bilaterian ancestor, respectively. For further information consult the legends for Figs. 3 and 4. Species abbreviations and accession numbers for each sequence are provided in Additional file 5. (PDF 530 kb)

Additional file 7: Phylogenetic tree illustrating the *tektin* gene diversity in spiralians. This phylogenetic analysis is focused on spiralian tektins including the full complement of the greatly expanded and divergent tektin gene complements of Platyhelminthes and the leech *H. robusta.* Both Bayesian and Maximum Likelihood analyses were performed using Mr. Bayes and RAxML, respectively. Bayesian tree is shown. Node support is shown for non-terminal nodes. Posterior probability values from Mr. Bayes are shown above each node and bootstrap values from RAxML are shown below each node. Diamonds indicate support less than 80%. An “X” under a node indicates this node was not recovered in the RAxML maximum likelihood tree. Tree was rooted with choanoflagellate Tektins. The planarian *S. mediterranea (Sme)* and the leech *H. robusta (Hr)* independently evolved an identical expanded *tektin* gene complement of ten *tektin* genes from the five ancestral spiralian *tektin* genes. Some Tektins were subsequently lost in the parasitic Platyhelminthes. Platyhelminthes species are highlighted with boxes. *S. mediterranea* and *H. robusta* are highlighted with red text. Species abbreviations and accession numbers for each sequence are provided in Additional file 5. (PDF 689 kb)

Additional file 8: Phylogenetic tree illustrating the *tektin* gene diversity in ecdysozoans. This phylogenetic analysis is focused on ecdysozoan tektins. Both Bayesian and Maximum Likelihood analyses were performed using Mr. Bayes and RAxML, respectively. Bayesian tree is shown. Node support is shown for non-terminal nodes. Posterior probability values from Mr. Bayes are shown above each node and bootstrap values from RAxML are shown below each node. Diamonds indicate support less than 80%. An “X” under a node indicates this node was not recovered in the RAxML maximum likelihood tree. Tree was rooted with choanoflagellate Tektin. Species abbreviations and accession numbers for each sequence are provided in Additional file 5. (PDF 659 kb)

Additional file 9: Comparison of this study vs. Nevers et al. [50] and Amos [20]. Comparison of findings of this study to phylogenetic analysis data by Nevers et al. [50] and Amos [20]. Species examined in both this study and at least one of the previous two are shown with the number of each Tektin class, and total number of Tektins found in each study. As the Nevers et al. [50] study used presence/absence data, a ‘+’ indicates presence while ‘0’ represents absence. Totals for Nevers et al. [50] are based on assumption that a ‘+’ is equal to one homolog. * indicates that different species from the same genus are compared. ** indicates different species from the same order or family are compared. *** indicates at least one Tektin did not group with any of the five recognized Tektins. † indicates that Nevers et al. [50] identified additional Tektin homologs that our study was unable to find. †† indicates the special case of human Tektins reported in Amos [20] (see Discussion). Green background indicates the previous study found the same number of Tektins as our study but with one or more misclassified. Red background indicates that the previous study found a different number of Tektins than our study. (PDF 660 kb)
