# Supplementary material for: Taxon-specific expansion and loss of tektins inform metazoan ciliary diversity
Source: BMC Evol Biol. 2019 Jan 31;19:40. doi: 10.1186/s12862-019-1360-0 (PMC6357514; doi:10.1186/s12862-019-1360-0)
Supplement: Supplementary file 1 — Comprehensive table of the tektin gene complement found in species included in this study (related to Table 1). The table shows all species examined in this study and their tektin gene complement with the exception of some teleost fish removed for redundancy. Species are listed according to four groups: (1) nonbilaterians, (2) spiralians, (3) ecdysozoans, and (4) deuterostomes. The tektin gene complement for each species is presented by total number of tektin genes within their genome in the right column, as well as the number of homologs found for each of the four bilaterian classes: Tektin-2, Tektin-1, Tektin-4, and Tektin-3/5 (in spiralians: Tektin-3/5A and 3/5B, in vertebrates: Tektin-3 and -5). The tektin gene complements for distinct inferred Last Common Ancestors (LCAs) are also shown. Gray boxes indicate metazoans that have maintained the ancestral tektin gene complement inferred for their clade (nonbilaterians, spiralians, ecdysozoans, or deuterostomes). Blue boxes indicate metazoans that have experienced duplications of tektin-3/5, but maintain single copies of tektin-1, − 2 and − 4. Pink boxes indicate the vertebrates that have lost tektin-5. Note that the four Tektin classes are bilaterian specific originating by ancient gene duplications from two nonbilaterian and one unicellular tektin gene(s). * indicates partial sequence left out of final analysis. ** indicates highly divergent long branch sequences after initial analyses and left out of final analysis. Yellow circles indicate key clades as follows. Unicellular Eukaryotes and Nonbilaterian Metazoans table: E = Eukaryota, O = Opisthokonta, H = Holozoa, A = Apoikozoa, M = Metazoa. Spiralian table: S = Spiralia, G = Gnathifera, R = Rouphozoa, M = Mesozoa, L = Lophotrochozoa. Ecdysozoan table: E = Ecdysozoa, A = Arthropoda. Deuterostome table: D = Deuterostomia, C = Chordata. Tree structures are based on – Eukaryotes: Burki 2014, Janouskevec et al. 2017, Torruella et al. 2015, and Budd and Jensen 2017 [7 [file 12862_2019_1360_MOESM1_ESM.pdf]

Eukaryote Tektins

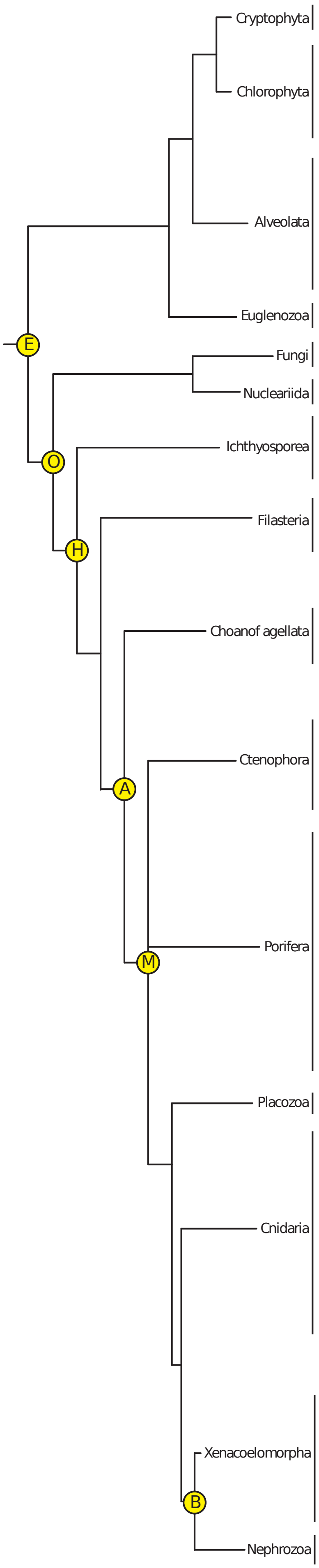

<table>
<tr><th>Species</th><th>Tektin-2</th><th>Tektin-1</th><th>Tektin-4</th><th>Tektin-3/5</th><th>Total</th></tr>
<tr><td>Eukaryotic LCA</td><td colspan="4">0</td><td>0</td></tr>
<tr><td>Guillardia theta</td><td colspan="4">1</td><td>1</td></tr>
<tr><td>Chlamydomonas reinhardtii</td><td colspan="4">1</td><td>1</td></tr>
<tr><td>Gonium pectorale</td><td colspan="4">1</td><td>1</td></tr>
<tr><td>Volvox carteri</td><td colspan="4">1</td><td>1</td></tr>
<tr><td>Symbiodinium microadriaticum</td><td colspan="4">0</td><td>0</td></tr>
<tr><td>Plasmodium falciporum</td><td colspan="4">0</td><td>0</td></tr>
<tr><td>Tetrahymena thermophila</td><td colspan="4">0</td><td>0</td></tr>
<tr><td>Paramecium tetraurelia</td><td colspan="4">0</td><td>0</td></tr>
<tr><td>Trypanosoma brucei</td><td colspan="4">0</td><td>0</td></tr>
<tr><td>Saccharomyces cerevisiae</td><td colspan="4">0</td><td>0</td></tr>
<tr><td>Parvularia atlantis</td><td colspan="4">0</td><td>0</td></tr>
<tr><td>Creolimax fragrantissima</td><td colspan="4">0</td><td>0</td></tr>
<tr><td>Sphaeroforma arctica</td><td colspan="4">0</td><td>0</td></tr>
<tr><td>Capsaspora owczarzaki</td><td colspan="4">0</td><td>0</td></tr>
<tr><td>Ministeria vibrans</td><td colspan="4">0</td><td>0</td></tr>
<tr><td>Apoikozoan LCA</td><td colspan="4">1</td><td>1</td></tr>
<tr><td>Monosiga ovata</td><td colspan="4">1</td><td>1</td></tr>
<tr><td>Salpingoeca rosetta</td><td colspan="4">1</td><td>1</td></tr>
<tr><td>Metazoan LCA</td><td>1</td><td colspan="3">1</td><td>2</td></tr>
<tr><td>Beroe abyssicola</td><td>1</td><td colspan="3">3</td><td>4</td></tr>
<tr><td>Mnemiopsis leidyi</td><td>1</td><td colspan="3">3</td><td>4</td></tr>
<tr><td>Pleurobrachia brachei</td><td>1</td><td colspan="3">3</td><td>4</td></tr>
<tr><td>Amphimedon queenslandica</td><td>1</td><td colspan="3">1</td><td>2</td></tr>
<tr><td>Ephydatia muelleri</td><td>1</td><td colspan="3">1</td><td>2</td></tr>
<tr><td>Haliclona amboinensis</td><td>0</td><td colspan="3">1</td><td>1</td></tr>
<tr><td>Haliclona tubifera</td><td>0</td><td colspan="3">1</td><td>1</td></tr>
<tr><td>Oscarella carmela</td><td>1</td><td colspan="3">1</td><td>2</td></tr>
<tr><td>Stylissa carteri</td><td>1</td><td colspan="3">1</td><td>2</td></tr>
<tr><td>Sycon ciliatum</td><td>1</td><td colspan="3">1</td><td>2</td></tr>
<tr><td>Trichoplax adhaerens</td><td>0</td><td colspan="3">0</td><td>0</td></tr>
<tr><td>Acropora digitifera</td><td>1</td><td colspan="3">2</td><td>3</td></tr>
<tr><td>Aurelia arita</td><td>1</td><td colspan="3">2*</td><td>3</td></tr>
<tr><td>Clytia hemisphaerica</td><td>1</td><td colspan="3">1</td><td>2</td></tr>
<tr><td>Exaiptasia pallida</td><td>1</td><td colspan="3">2</td><td>3</td></tr>
<tr><td>Hydra vulgaris</td><td>1</td><td colspan="3">1</td><td>2</td></tr>
<tr><td>Nematostella vectensis</td><td>1</td><td colspan="3">2</td><td>3</td></tr>
<tr><td>Bilaterian LCA</td><td>1</td><td>1</td><td colspan="2">1</td><td>3*</td></tr>
<tr><td>Convolutriloba macropyga</td><td>1</td><td>1</td><td colspan="2">1</td><td>3</td></tr>
<tr><td>Isodiametra pulchra</td><td>1</td><td>1</td><td colspan="2">1</td><td>3</td></tr>
<tr><td>Meara stichopi</td><td>1</td><td>1</td><td colspan="2">1</td><td>3</td></tr>
<tr><td>Xenoturbella bocki</td><td>1</td><td>1</td><td colspan="2">1</td><td>3</td></tr>
<tr><td>Neprhozoan LCA</td><td>1</td><td>1</td><td>1</td><td>1</td><td>4</td></tr>
</table>

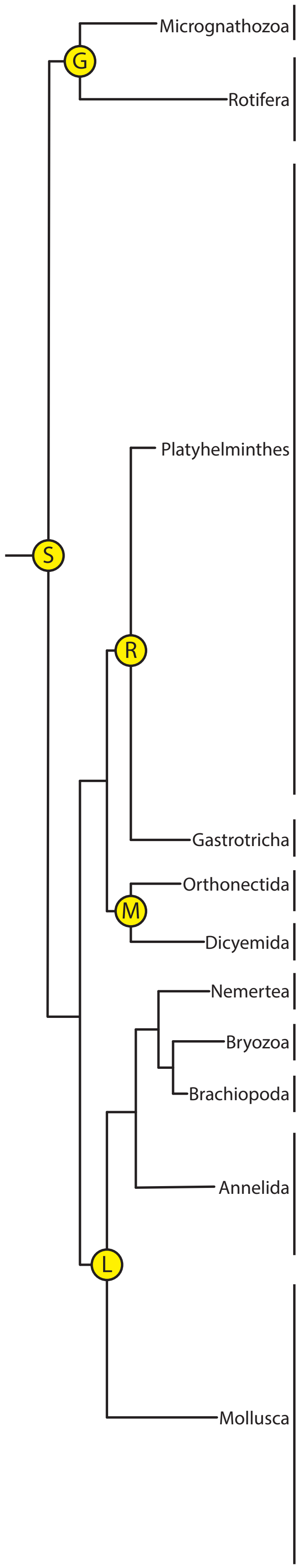

Spiralian Tektins

Species	Tektin-2	Tektin-1	Tektin-4	Tektin-3/5A	Tektin-3/5B	Total
Spiralian LCA	1	1	1	1	1	5
Limnognathia maerski	1	1	1	1	1	5
Adineta vaga	1	1	1	1	0	4
Brachionus calyciflorus	1	1	1	1	0	4
Macrostomum lignano	1	1	1	1	1	5
Prostheceraeus vittatus	1	1	1	2	2	7
Schmidtea mediterranea	2	2	2	3	1	10
Clonorchis sinensis	2	1	2	1	1	7
Opisthorchis viverrini	2	1	1	1	1	6
Schistosoma haematobium	2	1	1	1	1	6
Schistosoma japonicum	2*	1	1	1*	1*	6
Schistosoma mansoni	2	1	1	1	1	6
Echinococcus granulosus	2	1	1	1	0	5
Echinococcus multilocularis	2	1	1	1	0	5
Hymenolepis microstoma	2	1	1	1	0	5
Taenia asiatica	2	1	1	1	0	5
Taenia saginata	2	1	1	1**	0	5
Lepidodermella squamata	1	1	1	1	1	5
Intoshia linei	1**	0	0	1	1	3
Dicyema japonicum	1	1	0	0	0	2
Lineus longissimus	1	1	1	1	1	5
Membranipora membranacea	1	1	1	2	2	7
Lingula anatina	1	1	1	1	1	5
Capitella teleta	1	1	1	1	1	5
Helobdella robusta	2	2	2	3	1	10
Platynereis dumerilii	1	1	1	1	1	5
Crassostrea gigas	1	1	1	1	1	5
Pinctada fucata	1	1	1	1	1	5
Octopus bimaculoides	1	1	1	0	3	6
Aplysia californica	1	1	1	2	1	6
Biomphalaria glabrata	1*	1	1	2	1	6
Lottia gigantea	1	1	1	2	3	8

Ecdysozoan Tektins

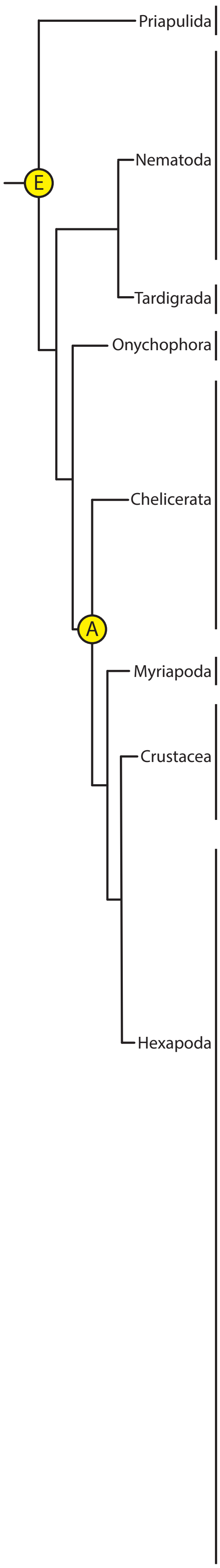

Species	Tektin-2	Tektin-1	Tektin-4	Tektin-3/5	Total
Ecdysozoan LCA	1	1	1	1	4
Priapulus caudatus	1	1	1	1	4
Caenorhabditis brenneri	0	0	1	0	1
Caenorhabditis briggsae	0	0	1	0	1
Caenorhabditis elegans	0	0	1	0	1
Caenorhabditis remanei	0	0	1	0	1
Toxocara canis	0	0	1	0	1
Hypsibius dujardini	1	1	0	0	2
Peripatopsis capensis	0	0	1	0	1
Limulus polyphemus	0	0	1*	0	1
Parasteatoda tepidariorum	1	1	1	0	3
Stegodyphus mimosarum	1	1	1	0	3
Ixodes scapularis	0	0	0	0	0
Sarcoptes scabiei	0	0	0	0	0
Tetranychus urticae	0	0	0	0	0
Strigamia maritima	1	1	1	1	4
Daphnia pulex	0	1	0	0	1
Lepeophtheirus salmonis	1*	1*	1*	1*	4
Argulus siamensis	1	1	1	1	4
Pediculus humanus corporis	2	1	1	1	5
Tribolium castaneum	1	1	1	1	4
Anopheles sinensis	1	1	1	1	4
Drosophila melanogaster	1	1	1	1	4
Cimex lectularius	1	1	1	1	4
Acromyrmex echinatior	1	1	1	2	5
Apis mellifera	1	1	1	2	5
Bombus terrestris	1	1	1	2	5
Camponotus floridanus	1	1	1	2	5
Megachile rotundata	1	1	1	2	5
Microplitis demolitor	1	1	1	2	5
Zootermopsis nevadensis	1	1	1	1	4
Danaus plexippus	2	1	3	1	7
Operophtera brumata	2	1	3	1**	7
Papilio machaon	2	1	3	1	7
Papilio xuthus	2	1	3	1	7

Deuterostome Tektins

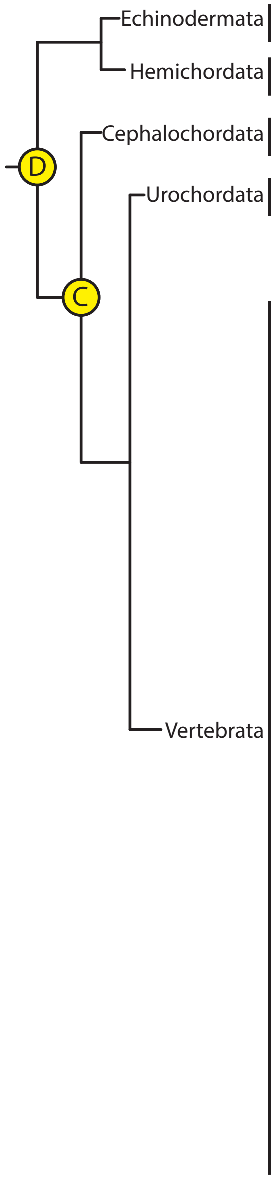

Species	Tektin-2	Tektin-1	Tektin-4	Tektin-3	Tektin-5	Total
Deuterostome LCA	1	1	1	1		4
Strongylocentrotus purpuratus	1	1	1	1		4
Saccoglossus kowalevskii	1	1	1	1		4
Branchiostoma floridae	1	1	1	1		4
Ciona intestinalis	1	1	1	1		4
Vertebrate LCA	1	1	1	1	1	5
Callorhincus milli	1	1	1	1	1	5
Lepisosteus oculatus	1	1	1	1	1	5
Clupea harengus	1	1	1	1	1	5
Danio rerio	1	1	1	1	0	4
Takifugu rubripes	1	1	1	1	0	4
Latimeria chalumnae	1	1	1	1	1	5
Ambystoma mexicanum	1	1	1	1	1	5
Notophthalmus viridescens	1	1	1	1	0	4
Xenopus laevis	1	1	1	1	0	4
Chrysemys picta belli	1	1	1	1	1	5
Anas platyrhynchos	1	1	1*	1	1	5
Gallus gallus	1	1	1	1	1	5
Ornithorhynchus anatinus	1	1	1*	1	1	5
Homo sapiens	1	1	1	1	1	5
Mus musculus	1	1	1	1	1	5
