# Supplementary material for: Taxon-specific expansion and loss of tektins inform metazoan ciliary diversity
Source: BMC Evol Biol. 2019 Jan 31;19:40. doi: 10.1186/s12862-019-1360-0 (PMC6357514; doi:10.1186/s12862-019-1360-0)
Supplement: Supplementary file 3 — Conservation and Divergence of Tektin proteins in bilaterians. Comparison of Tektin sequence conservation among selected species from spiralians (Mli, Cg, La, Pd), ecdysozoans (Tc, Bt, Dm, Pc, Sma), and deuterostomes (Hs, Gg, Bf, Sp, Sk). Percent identity shared at the amino acid level is given for each Tektin. Boxes are shaded according to degree of conservation with 30% identity or below being white, and 70% and above being the darkest blue. While spiralian and deuterostome exhibit high levels of sequence conservation at the amino acid level between Tektins of each class, ecdysozoans tend to have much lower sequence identity both when compared to spiralians and deuterostomes as well as when compared to other ecdysozoans. Exceptions are ecdysozoan Tektin-3/5 s that are generally higher conserved than other ecdysozoan Tektins belonging to the Tektin-1, − 2, and − 4 class. (PDF 628 kb) [file 12862_2019_1360_MOESM3_ESM.pdf]

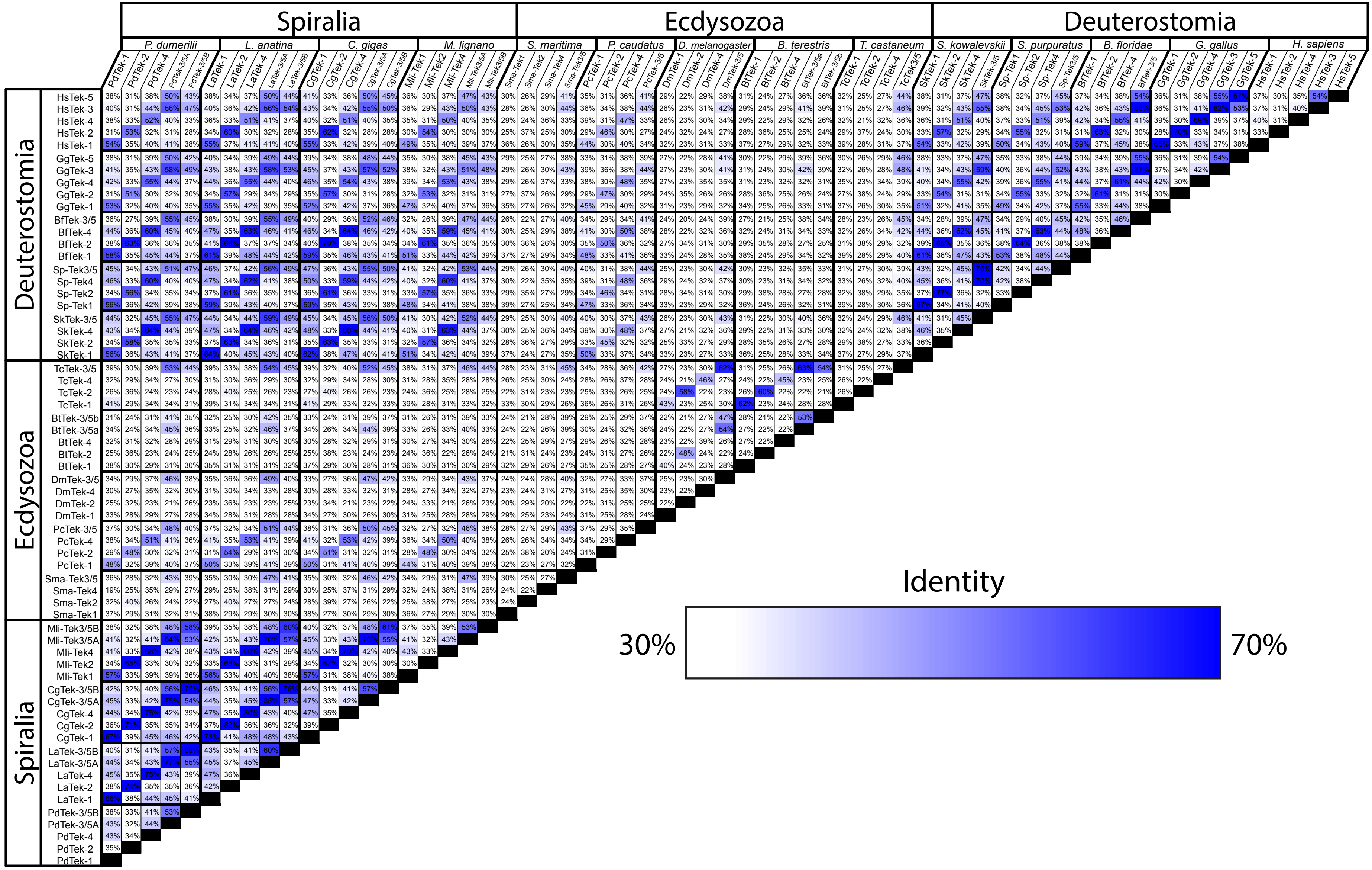

Spiralia
Ecdysozoa
Deuterostomia
P. dumerilii
L. anatina
C. gigas
M. lignano
S. maritima
P. caudatus
D. melanogaster
B. terestris
T. castaneum
S. kowalevskii
S. purpuratus
B. floridae
G. gallus
H. sapiens
PdTek-1
PdTek-2
PdTek-4
PdTek-3/5A
PdTek-3/5B
LaTek-1
LaTek-2
LaTek-4
LaTek-3/5A
LaTek-3/5B
CgTek-1
CgTek-2
CgTek-4
CgTek-3/5A
CgTek-3/5B
Mli-Tek1
Mli-Tek2
Mli-Tek4
Mli-Tek3/5A
Mli-Tek3/5B
Sma-Tek1
Sma-Tek2
Sma-Tek4
Sma-Tek3/5
PcTek-1
PcTek-2
PcTek-4
PcTek-3/5
DmTek-1
DmTek-2
DmTek-4
DmTek-3/5
BtTek-1
BtTek-2
BtTek-4
BtTek-3/5a
BtTek-3/5b
TcTek-1
TcTek-2
TcTek-4
TcTek-3/5
SkTek-1
SkTek-2
SkTek-4
SkTek-3/5
Sp-Tek1
Sp-Tek2
Sp-Tek4
Sp-Tek3/5
BfTek-1
BfTek-2
BfTek-4
BfTek-3/5
GgTek-1
GgTek-2
GgTek-4
GgTek-3
GgTek-5
HsTek-1
HsTek-2
HsTek-4
HsTek-3
HsTek-5
Identity
30%
70%
