# Supplementary material for: Taxon-specific expansion and loss of tektins inform metazoan ciliary diversity
Source: BMC Evol Biol. 2019 Jan 31;19:40. doi: 10.1186/s12862-019-1360-0 (PMC6357514; doi:10.1186/s12862-019-1360-0)
Supplement: Supplementary file 5 — List of species, source of sequences, and accession numbers of Tektins. List of all species examined in this study with names and accession numbers and/or other sequence identifiers for each Tektin identified. The source is given for the origin of each sequence obtained. * indicates partial sequence left out of final analysis. ** indicates highly divergent long branch sequences in initial analyses and left out of final analysis. Most sequences come from either NCBI or ENSEMBL. Other sequence sources include parasite.wormbase.org [79, 80], compagen.org [81], smedgd.stowers.org [82], neurobase.rc.ufl.edu, marinegenomics.oist.jp [83, 84], sandberg.cmb.ki.se [85], Mnemiopsis Genome Project Portal [86, 87], tardigrades.org, ambystoma.org [88, 89], or transcriptomic data provided by Dr. Andreas Hejnol used in Cannon et al, 2016 [62]. (DOCX 44 kb) [file 12862_2019_1360_MOESM5_ESM.docx]

Species

Sequence Accession Database

1. Acropora digitifera

Ad_Tek1/4/3/5A XP_015762460.1 NCBI

Ad_Tek1/4/3/5B aug_v2a.11765.t1 marinegenomics.oist.jp

Ad_Tek2 adi_EST_assem_5228 marinegenomics.oist.jp

2. Acromyrmex echinatior

Ae_Tek1 XP_011067874.1 NCBI

Ae_Tek2 XP_011062036.1 NCBI

Ae_Tek4 XP_011056331.1 NCBI

Ae_Tek3/5a XP_011069058.1 NCBI

Ae_Tek3/5b XP_011052779.1 NCBI

3. Adineta vaga

Av_Tek1 GSADVT00012708001 Ensembl

Av_Tek2 GSADVT00015520001 Ensembl

Av_Tek4 GSADVT00016725001 Ensembl

Av_Tek3/5A GSADVT00000148001 Ensembl

4. Ambystoma mexicanum

Amb_Tek1 isotig131207 ambystoma.org

Amb_Tek2 isotig216311 ambystoma.org

Amb_Tek4 isotig229956 ambystoma.org

Amb_Tek3 isotig213987 ambystoma.org

Amb_Tek5 isotig314383 and isotig131208 ambystoma.org

5. Amphimedon queenslandica

Aq_Tek1/4/3/5 XP_003389443.1 NCBI

Aq_Tek2 XP_003384329.1 NCBI

6. Anas platyrhynchos

Ap_Tek1 XP_012956465.1 NCBI

Ap_Tek2 XP_005025388.1 NCBI

Ap_Tek4* EOB07148.1 NCBI

Ap_Tek3 XP_005025855.1 NCBI

Ap_Tek5 XP_005027557.1 NCBI

7. Anopheles sinensis

As_Tek1 KFB53032.1 NCBI

As_Tek2 KFB42877.1 NCBI

As_Tek4 KFB35854.1 NCBI

As_Tek3/5 KFB37198.1 NCBI

8. Apis mellifera

Ame_Tek1 XP_397385.2 NCBI

Ame_Tek2 XP_001122977.1 NCBI

Ame_Tek4 XP_392103.2 NCBI

Ame_Tek3/5a XP_395193.1 NCBI

Ame_Tek3/5b XP_006567401.1 NCBI

9. Aplysia californica

Ac_Tek1 XP_005109632.1 NCBI

Ac_Tek2 XP_005098539.1 NCBI

Ac_Tek4 XP_005092496.1 NCBI

Ac_Tek3/5A1 XP_012939623.1 NCBI

Ac_Tek3/5A2 XP_012942396.1 NCBI

Ac_Tek3/5B XP_012945184.1 NCBI

10. Argulus siamensis

Ars_Tek1 Locus_14981.0_Transcript_1 Dr. Andreas Hejnol

Ars_Tek2 Locus 3041.0_Transcript_1 Dr. Andreas Hejnol

Ars_Tek4 Locus_19539.0_Transcript_10 Dr. Andreas Hejnol

Ars_Tek3/5 Locus_15466.0_Transcript_3 Dr. Andreas Hejnol

11. Astyanax mexicanus

Am_Tek3 XP_007248806.1 NCBI

12. Aurelia arita

Aa_Tek1/4/3/5A 03_aurelia_rc_finalASM_10226 compagen.org

Aa_Tek1/4/3/5B* 03_aurelia_rc_finalASM_10166 compagen.org

Aa_Tek2 03_aurelia_rc_finalASM_8448 compagen.org

13. Beroe abyssicola

Ba_Tek1/4/3/5a 12146574 neurobase.rc.ufl.edu

Ba_Tek1/4/3/5b 12126750 neurobase.rc.ufl.edu

Ba_Tek1/4/3/5c 12137034 neurobase.rc.ufl.edu

Ba_Tek2 12133366 neurobase.rc.ufl.edu

14. Biomphalaria glabrata

Bg_Tek1* XP_013064715.1 NCBI

Bg_Tek2 XP_013074221.1 NCBI

Bg_Tek4 XP_013090287.1 NCBI

Bg_Tek3/5A1 XP_013095021.1 NCBI

Bg_Tek3/5A2 XP_013086660.1 NCBI

Bg_Tek3/5B XP_013079790.1 NCBI

15. Bombus terrestris

Bt_Tek1 XP_003394478.1 NCBI

Bt_Tek2 XP_003394478.1 NCBI

Bt_Tek4 XP_003402172.1 NCBI

Bt_Tek3/5a XP_003401272.1 NCBI

Bt_Tek3/5b XP_003397505.1 NCBI

16. Brachionus calyciflorus

Bc_Tek1 c26206_g1_i1 Dr. Andreas Hejnol

Bc_Tek2 c20013_g1_i1 Dr. Andreas Hejnol

Bc_Tek4 c16631_g1_i1 Dr. Andreas Hejnol

Bc_Tek3/5A c16406_g1_iq Dr. Andreas Hejnol

17. Branchiostoma floridae

Bf_Tek1 XP_002590719.1 NCBI

Bf_Tek2 XP_002591791.1 NCBI

Bf_Tek4 XP_002590153.1 NCBI

Bf_Tek3/5 Brafl1 126663 JGI

18. Caenorhabditis brenneri

Cbre_Tek4** EGT30453.1 NCBI

19. Caenorhabditis briggsae

Cbri_Tek4** XP_002644456.1 NCBI

20. Caenorhabditis elegans

Ce_Tek4** NP_508689.1 NCBI

21. Caenorhabditis remanei

Cr_Tek4** XP_003118040.1 NCBI

22. Callorhinchus milli

Cm_Tek1 XP_007894703.1 NCBI

Cm_Tek2 XP_007906940.1 NCBI

Cm_Tek4 XP_007908645.1 NCBI

Cm_Tek3 XP_007886641.1 NCBI

Cm_Tek5 XP_007903082.1 NCBI

23. Camponotus floridanus

Cf_Tek1 XP_011251049.1 NCBI

Cf_Tek2 XP_011266903.1 NCBI

Cf_Tek4 XP_011258069.1 NCBI

Cf_Tek3/5a XP_011251575.1 NCBI

Cf_Tek3/5b XP_011251840.1 NCBI

24. Capitella teleta

Ct_Tek1 ELT88431.1 NCBI

Ct_Tek2 ELU06439.1 NCBI

Ct_Tek4 ELT92180.1 NCBI

Ct_Tek3/5A ELU07223.1 NCBI

Ct_Tek3/5B ELT96237.1 NCBI

25. Chlamydomonas reinhardtii

Chr_Tek2/1/4/3/5 BAC77347.1 NCBI

26. Chrysemys picta belli

Cpb_Tek1 XP_005298983.1 NCBI

Cpb_Tek2 XP_005298433.1 NCBI

Cpb_Tek4 XP_008162984.1 NCBI

Cpb_Tek3 XP_008162057.1 NCBI

Cpb_Tek5 XP_005306767.1 NCBI

27. Cimex lectularius

Cl_Tek1 XP_014261958.1 NCBI

Cl_Tek2 XP_014244697.1 NCBI

Cl_Tek4 XP_014250312.1 NCBI

Cl_Tek3/5 XP_014248255.1 NCBI

28. Ciona intestinalis

Ci_Tek1 XP_002130466.1 NCBI

Ci_TekB NP_001027645.1 NCBI

Ci_Tek4 NP_001027644.1 NCBI

Ci_Tek3/5 XP_002129626.1 NCBI

29. Clonorchis sinensis

Cs_Tek1 GAA51618.1 NCBI

Cs_Tek2a GAA37293.1 NCBI

Cs_Tek2b GAA54519.1 NCBI

Cs_Tek4a GAA33438.1 NCBI

Cs_Tek4b GAA27704.2 NCBI

Cs_Tek3/5A GAA56634.1 and GAA57313.1 NCBI

Cs_Tek3/5B GAA54568.1 NCBI

30. Clupea harengus

Ch_Tek1 XP_012684301.1 NCBI

Ch_Tek2 XP_012684937.1 NCBI

Ch_Tek4 XP_012678497.1 NCBI

Ch_Tek3 XP_012669882.1 NCBI

Ch_Tek5 XP_012682720.1 NCBI

31. Clytia hemisphaerica

Che_Tek1/4/3/5A unigene004679 compagen.org

Che_Tek2 unigene005322 compagen.org

32. Convolutriloba macropyga

Cma_Tek1 Cmac.rna.tri.1619.1 Dr. Andreas Hejnol

Cma_Tek2 Cmac.rna.tri.906.1 Dr. Andreas Hejnol

Cma_Tek4 Cmac.rna.tri.884.1 Dr. Andreas Hejnol

33. Crassostrea gigas

Cg_Tek1 EKC40190.1 NCBI

Cg_Tek2 EKC18085.1 NCBI

Cg_Tek4 XP_011416098.1 NCBI

Cg_Tek3/5A XP_011450983.1 NCBI

Cg_Tek3/5B XP_011415439.1 NCBI

34. Danaus plexippus

Dpl_Tek1 EHJ65372.1 NCBI

Dpl_Tek2a EHJ70734.1 NCBI

Dpl_Tek2b EHJ75592.1 NCBI

Dpl_Tek4a EHJ78645.1 NCBI

Dpl_Tek4b EHJ78646.1 NCBI

Dpl_Tek4c EHJ73640.1 NCBI

Dpl_Tek3/5 EHJ78859.1 NCBI

35. Danio rerio

Dr_Tek1 XP_009290074.1 NCBI

Dr_Tek2 NP_001017432.3 NCBI

Dr_Tek4 NP_001139162.1 NCBI

Dr_Tek3 XP_701169.2 NCBI

36. Daphnia pulex

Dp_Tek1 EFX80868.1 NCBI

37. Dicyema japonicum

Dj_TekC BAF46876.1 NCBI

Dj_TekB BAF46874.1 NCBI

38. Drosophila melanogaster

Dm_TekC NP_523940.2 NCBI

Dm_Tek2 CAL25869.1 NCBI

Dm_TekA NP_523577.1 NCBI

Dm_Tek3/5 NP_728442.1 NCBI

39. Echinococcus granulosus

Eg_Tek1 CDS20170.1 NCBI

Eg_Tek2a EUB56753.1 NCBI

Eg_Tek2b CDS20562.1 NCBI

Eg_Tek4a EUB62280.1 NCBI

Eg_Tek3/5A CDS20543.1 NCBI

40. Echinococcus multilocularis

Emu_Tek1 CDI98515.1 NCBI

Emu_Tek2a CDI97781.1 NCBI

Emu_Tek2b CDS43478.1 NCBI

Emu_Tek4a CUT99135.1 NCBI

Emu_Tek3/5A CDS43459.1 NCBI

41. Ephydatia muelleri

Em_Tek1/4/3/5 m.17284 compagen.org

Em_Tek2 m.18240 compagen.org

42. Esox lucius

El_Tek3 XP_010886950.1 NCBI

43. Exaiptasia pallida

Ep_Tek1/4/3/5A KXJ29004.1 NCBI

Ep_Tek1/4/3/5B KXJ24946.1 NCBI

Ep_Tek2 KXJ13215.1 NCBI

44. Gallus gallus

Gg_Tek1 XP_004946886.1 NCBI

Gg_Tek2 XP_417774.3 NCBI

Gg_Tek4 XP_414831.1 NCBI

Gg_Tek3 XP_415594.2 NCBI

Gg_Tek5 XP_414934.3 NCBI

45. Gonium pectorale

Gp_Tek2/1/4/3/5 KXZ52634.1 NCBI

46. Guillardia theta

Gt_Tek2/1/4/3/5 XP_005840601.1 NCBI

47. Haliclona amboinensis

Ha_Tek1/4/3/5 mm.2839 compagen.org

48. Haliclona tubifera

Ht_Tek1/4/3/5 m.9979 compagen.org

49. Hypsibius dujardini

Hd_Tek1 nHd.2.3.1.t18513-RA tardigrades.org

Hd_Tek2 nHd.2.3.1.t03698-RA tardigrades.org

50. Helobdella robusta

Hr_Tek1a XP_009029392.1 NCBI

Hr_Tek1b XP_009027853.1 NCBI

Hr_Tek2a XP_009031973.1 NCBI

Hr_Tek2b XP_009012149.1 NCBI

Hr_Tek4a XP_009030133.1 NCBI

Hr_Tek4b XP_009019508.1 NCBI

Hr_Tek3/5A1 XP_009023847.1 NCBI

Hr_Tek3/5A2 XP_009030119.1 NCBI

Hr_Tek3/5A3 XP_009028491.1 NCBI

Hr_Tek3/5B XP_009022344.1 NCBI

51. Homo sapiens

Hs_Tek1 NP_444515.1 NCBI

Hs_Tek2 NP_055281.2 NCBI

Hs_Tek4 NP_653306.1 NCBI

Hs_Tek3 NP_114104.1 NCBI

Hs_Tek5 NP_653275.1 NCBI

52. Hydra vulgaris

Hv_Tek1/4/3/5 XP_012561733.1 NCBI

Hv_Tek2 XP_002158675.2 NCBI

53. Hymenolepis microstoma

Hmi_Tek1 CDS26520.1 NCBI

Hmi_Tek2a CDS25896.1 NCBI

Hmi_Tek2b CDS30467.1 NCBI

Hmi_Tek4a CDS28440.1 NCBI

Hmi_Tek3/5 CDS30499.1 NCBI

54. Intoshia linei

Il_Tek1L** OAF66009.1 NCBI

Il_Tek2 OAF66679.1 NCBI

Il_Tek3/5A OAF65440.1 NCBI

Il_Tek3/5B OAF65547.1 NCBI

55. Isodiametra pulchra

Ipu_Tek1 Ipul.rna.tri.1193.1 Dr. Andreas Hejnol

Ipu_Tek2 Ipul.rna.tri.641.1 Dr. Andreas Hejnol

Ipu_Tek4 Ipul.rna.tri.939.1 Dr. Andreas Hejnol

56. Latimeria chalumnae

Lc_Tek1 XP_006014220.1 NCBI

Lc_Tek2 XP_014352741.1 NCBI

Lc_Tek4 XP_005997853.1 NCBI

Lc_Tek3 XP_005989354.1 NCBI

Lc_Tek5 XP_006007946.1 NCBI

57. Lepidodermella squamata

Lsq_Tek1 Lsqu.rna.tri.21850.1 Dr. Andreas Hejnol

Lsq_Tek2 Lsqu.rna.tri.35299.1 Dr. Andreas Hejnol

Lsq_Tek4 Lsqu.rna.tri.23979.1 Dr. Andreas Hejnol

Lsq_Tek3/5A Lsqu.rna.tri.46354.1 Dr. Andreas Hejnol

Lsq_Tek3/5B Lsqu.rna.tri.36597.2 Dr. Andreas Hejnol

58. Lineus longissimus

Ll_Tek1 Locus_17751.0_Transcript_2 Dr. Andreas Hejnol

Ll_Tek2 Locus_49478.0_Transcript_1 Dr. Andreas Hejnol

Ll_Tek4 Locus_51495.0_Transcript_2 Dr. Andreas Hejnol

Ll_Tek3/5A Locus_46321.0_Transcript_6 Dr. Andreas Hejnol

Ll_Tek3/5B Locus_45753.0_Transcript_7 Dr. Andreas Hejnol

59. Lingula anatina

La_Tek1 XP_013403743.1 NCBI

La_Tek2 XP_013390065.1 NCBI

La_Tek4 XP_013399215.1 NCBI

La_Tek3/5A XP_013387004.1 NCBI

La_Tek3/5B XP_013384963.1 NCBI

60. Limnognathia maerski

Lm_Tek1 Locus_17751.0_Transcript_2 Dr. Andreas Hejnol

Lm_Tek2 Locus_11742.0_Transcript_2 Dr. Andreas Hejnol

Lm_Tek4 Locus_15132.0_Transcript_2 Dr. Andreas Hejnol

Lm_Tek3/5A Locus_14155.0_Transcript_1 Dr. Andreas Hejnol

Lm_Tek3/5B Locus_17238.0_Transcript_1 Dr. Andreas Hejnol

61. Lepeophtheirus salmonis

Ls_Tek1* EMLSAG00000002130 Ensembl

Ls_Tek2* EMLSAG00000007697 Ensembl

Ls_Tek4* EMLSAG00000008536 Ensembl

Ls_Tek3/5* EMLSAG00000000573 Ensembl

62. Lepisosteus oculatus

Lo_Tek1* XP_015222843.1 NCBI

Lo_Tek2 XP_006631366.1 NCBI

Lo_Tek4 XP_006637103.1 NCBI

Lo_Tek3 XP_006635248.1 NCBI

Lo_Tek5 XP_015215582.1 NCBI

63. Limulus polyphemus

Lp_Tek4* XP_013776902 NCBI

64. Lottia gigantea

Lg_Tek1 XP_009053992.1 NCBI

Lg_Tek2 XP_009060972.1 NCBI

Lg_Tek4 XP_009060179.1 NCBI

Lg_Tek3/5A1 XP_009064336.1 NCBI

Lg_Tek3/5A2 XP_009064337.1 NCBI

Lg_Tek3/5B1 XP_009048507.1 NCBI

Lg_Tek3/5B2 XP_009048510.1 NCBI

Lg_Tek3/5B3 XP_009048516.1 NCBI

65. Macrostomum lignano

Mli_Tek1 maker-uti_cns_0003731-snap-gene-0.6 parasite.wormbase.org

Mli_Tek2 maker-uti_cns_0003921-snap-gene-0.8 parasite.wormbase.org

Mli_Tek3/5A maker-uti_cns_0001415-snap-gene-0.5 parasite.wormbase.org

Mli_Tek3/5B maker-uti_cns_0004589-snap-gene-0.3 parasite.wormbase.org

Mli_Tek4 maker-uti_cns_0009859-snap-gene-0.6 parasite.wormbase.org

66. Meara stichopi

Mst_Tek1 Msti.rna.tri.4700.1 Dr. Andreas Hejnol

Mst_Tek2 Msti.rna.tri.4881.1 Dr. Andreas Hejnol

Mst_Tek4 Msti.rna.tri.4063.1 Dr. Andreas Hejnol

67. Megachile rotundata

Mr_Tek1 XP_003703991.1 NCBI

Mr_Tek2 XP_003705658.1 NCBI

Mr_Tek4 Mrot01696 hymenopteragenome.org

Mr_Tek3/5a XP_003708662.1 NCBI

Mr_Tek3/5b XP_012150848.1 NCBI

68. Membranipora membranacea

Mme_Tek1 Mmem.rna.tri.8544.1 Dr. Andreas Hejnol

Mme_Tek2 Mmem.rna.tri.3520.1 Dr. Andreas Hejnol

Mme_Tek4 Mmem.rna.tri.7108.1 Dr. Andreas Hejnol

Mme_Tek3/5A1 Mmem.rna.tri.9342.1 Dr. Andreas Hejnol

Mme_Tek3/5A2 Mmem.rna.tri.3520.1 Dr. Andreas Hejnol

Mme_Tek3/5B1 Mmem.rna.tri.12517.1 Dr. Andreas Hejnol

Mme_Tek3/5B2 Mmem.rna.tri.19484.1 Dr. Andreas Hejnol

69. Microplitis demolitor

Md_Tek1 XP_008546540.1 NCBI

Md_Tek2 XP_008558946.1 NCBI

Md_Tek4 XP_014298184.1 NCBI

Md_Tek3/5a XP_008548432.1 NCBI

Md_Tek3/5b XP_008543201.2 NCBI

70. Mnemiopsis leidyi

Ml_Tek1/4/3/5a ML305512a Mnemiopsis genome project portal

Ml_Tek1/4/3/5b ML01493a Mnemiopsis genome project portal

Ml_Tek1/4/3/5c ML047948a Mnemiopsis genome project portal

Ml_Tek2 12510233 neurobase.rc.ufl.edu

71. Monosiga ovata

Mo_Tek2/1/4/3/5 CL3843Contig1 compagen.org

72. Mus musculus

Mm_Tek1 EDL12674.1 NCBI

Mm_Tek2 NP_036032.2 NCBI

Mm_Tek4 NP_082227.1 NCBI

Mm_Tek3 NP_081936.1 NCBI

Mm_Tek5 ADD80740.1 NCBI

73. Nematostella vectensis

Nv_Tek1/4/3/5A XP_001631355.1 NCBI

Nv_Tek1/4/3/5B XP_001632744.1 NCBI

Nv_Tek2 XP_001629420.1 NCBI

74. Notophthalmus viridescens

Nvi_Tek1 comp100970_c0_seq1 sandberg.cmb.ki.se

Nvi_Tek2 comp1429794_c0_seq1 sandberg.cmb.ki.se

Nvi_Tek4 comp108093_c0_seq1 sandberg.cmb.ki.se

Nvi_Tek3 comp152641_c0_seq1 sandberg.cmb.ki.se

75. Octopus bimaculoides

Ob_Tek1 XP_014786778.1 NCBI

Ob_Tek2 XP_014774177.1 NCBI

Ob_Tek4 XP_014784462.1 NCBI

Ob_Tek3/5A1 XP_014784682.1 NCBI

Ob_Tek3/5A2 XP_014788021.1 NCBI

Ob_Tek3/5A3 XP_014774959.1 NCBI

76. Operophtera brumata

Obr_Tek1 KOB68614.1 NCBI

Obr_Tek2a KOB68231.1 NCBI

Obr_Tek2b KOB76247.1 NCBI

Obr_Tek4a KOB75888.1 NCBI

Obr_Tek4b KOB75887.1 NCBI

Obr_Tek4c KOB72936.1 NCBI

Obr_Tek3/5** KOB68386.1 NCBI

77. Opisthorchis viverrini

Ov_Tek1 XP_009167541.1 NCBI

Ov_Tek2a XP_009171112.1 NCBI

Ov_Tek2b XP_009170825.1 NCBI

Ov_Tek4a XP_009163119.1 NCBI

Ov_Tek3/5A XP_009164681.1 NCBI

Ov_Tek3/5B XP_009163742.1 NCBI

78. Oreochromis niloticus

On_Tek3 XP_003453437.1 NCBI

79. Ornithorhynchus anatinus

Oa_Tek1 XP_001511748.1 NCBI

Oa_Tek2 XP_007668706.1 NCBI

Oa_Tek4* XP_001520907.1 NCBI

Oa_Tek3 XP_001506232.1 NCBI

Oa_Tek5 XP_001505552.1 NCBI

80. Oscarella carmela

Oc_Tek1/4/3/5 m.309007 compagen.org

Oc_Tek2 m.16661 compagen.org

81. Papilio machaon

Pm_Tek1 XP_014360507.1 NCBI

Pm_Tek2a XP_014357368.1 NCBI

Pm_Tek2b KPJ16585.1 NCBI

Pm_Tek4a KPJ20100.1 NCBI

Pm_Tek4b XP_014358160.1 NCBI

Pm_Tek4c KPJ18494.1 NCBI

Pm_Tek3/5 XP_014368606.1 NCBI

82. Papilio xuthus

Px_Tek1 XP_013171663.1 NCBI

Px_Tek2a XP_013173813.1 NCBI

Px_Tek2b XP_013172105.1 NCBI

Px_Tek4a XP_013165499.1 NCBI

Px_Tek4b XP_013165487.1 NCBI

Px_Tek4c KPI93218.1 NCBI

Px_Tek3/5 KPJ02176.1 NCBI

83. Parasteatoda tepidariorum

Pt_Tek1 XP_015912205.1 NCBI

Pt_Tek2 XP_015930507.1 NCBI

Pt_Tek4 XP_015914488.1 NCBI

84. Pediculus humanus corporis

Phc_Tek1 XP_002423983.1 NCBI

Phc_Tek2a XP_002430254.1 NCBI

Phc_Tek2b XP_002430261.1 NCBI

Phc_Tek4 XP_002429992.1 NCBI

Phc_Tek3/5 XP_002427060.1 NCBI

85. Peripatopsis capensis

Pcap_Tek4 c85140_g1_i1 Dr. Andreas Hejnol

86. Pinctada fucata

Pf_Tek1 pfu_aug2.0_1553.1_11730.t1 marinegenomics.oist.jp

Pf_Tek2 pfu_aug2.0_1190.1_01460.t1 marinegenomics.oist.jp

Pf_Tek4 pfu_aug2.0_716.1_21085.t1 marinegenomics.oist.jp

Pf_Tek3/5A pfu_aug2.0_1874.1_15289.t1 marinegenomics.oist.jp

Pf_Tek3/5B pfu_aug2.0_389.1_30702.t1 marinegenomics.oist.jp

87. Platynereis dumerilii

Pd_Tek1

Pd_Tek2

Pd_Tek4

Pd_Tek3/5A

Pd_Tek3/5B

88. Pleurobrachia brachei

Pb_Tek1/4/3/5a 2642861 neurobase.rc.ufl.edu

Pb_Tek1/4/3/5b 2664729 neurobase.rc.ufl.edu

Pb_Tek1/4/3/5c 2645004, 2645006, 2647858, 2643919, 2654206 neurobase.rc.ufl.edu

Pb_Tek2 2640062 neurobase.rc.ufl.edu

89. Priapulus caudatus

Pc_Tek1 XP_014666311.1 NCBI

Pc_Tek2 XP_014674115.1 NCBI

Pc_Tek4 XP_014664115.1 NCBI

Pc_Tek3/5 XP_014675800.1 and XP_014675801.1 NCBI

90. Prostheceraeus vittatus

Pv_Tek1 Pvit.rna.tri.844.1 Dr. Andreas Hejnol

Pv_Tek2 Pvit.rna.tri.756.1 Dr. Andreas Hejnol

Pv_Tek4 Pvit.rna.tri.743.1 Dr. Andreas Hejnol

Pv_Tek3/5A1 Pvit.rna.tri.898.1 Dr. Andreas Hejnol

Pv_Tek3/5A2 Pvit.rna.tri.4944.1 Dr. Andreas Hejnol

Pv_Tek3/5B1 Pvit.rna.tri.1680.1 Dr. Andreas Hejnol

Pv_Tek3/5B2 Pvit.rna.tri.2719.1 Dr. Andreas Hejnol

91. Saccoglossus kowalevskii

Sk_Tek1 XP_002734203.1 NCBI

Sk_Tek2 XP_002734332.1 NCBI

Sk_Tek4 XP_006812879.1 NCBI

Sk_Tek3/5 XP_002732125.2 NCBI

92. Salmo salar

Ss_Tek3 XP_014035309.1 NCBI

93. Salpingoeca rosetta

Sr_Tek2/1/4/3/5 XP_004996082.1 NCBI

94. Schistosoma haematobium

Sch_Tek1 XP_012794787.1 NCBI

Sch_Tek2a XP_012793974.1 NCBI

Sch_Tek2b XP_012800500.1 NCBI

Sch_Tek4b XP_012794579.1 NCBI

Sch_Tek3/5A XP_012793419.1 and XP_012793420.1 NCBI

Sch_Tek3/5B XP_012792113.1 NCBI

95. Schistosoma japonicum

Scj_Tek1 CAX69671.1 NCBI

Scj_Tek2a CAX73548.1 NCBI

Scj_Tek2b* AAX26067.2 NCBI

Scj_Tek4b CAX70198.1 NCBI

Scj_Tek3/5A* AAX30175.1 NCBI

Scj_Tek3/5B* AAW25793.1 NCBI

96. Schistosoma mansoni

Scm_Tek1 CCD75138.1 NCBI

Scm_Tek2a CCD80732.1 NCBI

Scm_Tek2b CAZ36106.2 NCBI

Scm_Tek4b CCD82121.1 NCBI

Scm_Tek3/5A CCD80649.1 and CCD80650.1 NCBI

Scm_Tek3/5B CCD78298.1 NCBI

97. Schmidtea mediterranea

Sme_Tek1a SMU15011967 SmedGD.stowers.org

Sme_Tek1b SMU15003266 SmedGD.stowers.org

Sme_Tek2a SMU15038167 SmedGD.stowers.org

Sme_Tek2b SMU15018661 SmedGD.stowers.org

Sme_Tek4a SMU15038883 SmedGD.stowers.org

Sme_Tek4b SMU15039333 SmedGD.stowers.org

Sme_Tek3/5A1 SMU15029848 SmedGD.stowers.org

Sme_Tek3/5A2 SMU15000846 SmedGD.stowers.org

Sme_Tek3/5A3 SMU15000154 SmedGD.stowers.org

Sme_Tek3/5B SMU15001292 SmedGD.stowers.org

98. Stegodyphus mimosarum

Smi_Tek1 EG:KK120086 (genome contig, inferrd) Ensembl

Smi_Tek2 EG:KK113373 (genome contig,inferred) Ensembl

Smi_Tek4 KFM83011.1 NCBI

99. Strigamia maritima

Sma_Tek1 SMAR009243-PA Ensembl

Sma_Tek2 SMAR011315-PA Ensembl

Sma_Tek4 SMAR013151-PA Ensembl

Sma_Tek3/5 SMAR001297-PA Ensembl

100. Strongylocentrotus purpuratus

Sp_TekC1 NP_999788.1 NCBI

Sp_TekB1 NP_999789.1 NCBI

Sp_TekA1 XP_011667423.1 NCBI

Sp_Tek3/5 XP_791206.2 NCBI

101. Stylissa carteri

Sc_Tek1/4/3/5 maker-SC_scaffold5351-snap-gene-0.14-mRNA-1 compagen.org

Sc_Tek2 maker-SC_scaffold4756-snap-gene-0.31-mRNA-1 compagen.org

102. Sycon ciliatum

Sci_Tek1/4/3/5 scpid77689 compagen.org

Sci_Tek2 scpid51362 compagen.org

103. Taenia asiatica

Taa_Tek1 TASK_0000215101 parasite.wormbase.org

Taa_Tek2a TASK_0000732201 parasite.wormbase.org

Taa_Tek2b TASK_0000880201 parasite.wormbase.org

Taa_Tek4a TASK_0000228801 parasite.wormbase.org

Taa_Tek3/5A TASK_0000434301 parasite.wormbase.org

104. Taenia saginata

Tas_Tek1 OCK41512.1 NCBI

Tas_Tek2a OCK38190.1 NCBI

Tas_Tek2b OCK35575.1 NCBI

Tas_Tek4a OCK39319.1 NCBI

Tas_Tek3/5A** OCK35556.1 NCBI

105. Takifugu rubripes

Tr_Tek1 XP_011607045.1 NCBI

Tr_Tek2 XP_003969284.1 NCBI

Tr_Tek4 XP_003972094.1 NCBI

Tr_Tek3 XP_003961064.1 NCBI

106. Toxocara canis

Tca_Tek4* KHN70608.1 NCBI

107. Tribolium castaneum

Tc_Tek1 XP_972983.2 NCBI

Tc_Tek2 XP_967107.1 NCBI

Tc_Tek4 XP_973164.1 NCBI

Tc_Tek3/5 XP_974692.1 NCBI

108. Volvox carteri

Vc_Tek2/1/4/3/5 XP_002957917.1 NCBI

109. Xenopus laevis

Xl_Tek1 NP_001085226.1 NCBI

Xl_Tek2 NP_001079467.1 NCBI

Xl_Tek4 NP_001088802.1 NCBI

Xl_Tek3 NP_001079857.1 NCBI

110. Xenoturbella bocki

Xbo_Tek1 Xboc.rna.tri.4156.1 Dr. Andreas Hejnol

Xbo_Tek2 Xboc.rna.tri.3411.1 Dr. Andreas Hejnol

Xbo_Tek4 Xboc.rna.tri.3135.1 Dr. Andreas Hejnol

111. Zootermopsis nevadensis

Zn_Tek1 KDR20069.1 NCBI

Zn_Tek2 KDR14917.1 NCBI

Zn_Tek4 KDR13451.1 NCBI

Zn_Tek3/5 KDR12673.1 NCBI
