# Supplementary material for: Taxon-specific expansion and loss of tektins inform metazoan ciliary diversity
Source: BMC Evol Biol. 2019 Jan 31;19:40. doi: 10.1186/s12862-019-1360-0 (PMC6357514; doi:10.1186/s12862-019-1360-0)
Supplement: Supplementary file 6 — Comprehensive phylogenetic tree of the tektin gene family (related to Figs. 3 and 4). This comprehensive phylogenetic analysis includes species representing all major metazoan lineages, choanoflagellates and algae. Both Bayesian and Maximum Likelihood analyses were performed using Mr. Bayes and RAxML, respectively. Bayesian tree is shown. Node support is shown for non-terminal nodes. Posterior probability values from Mr. Bayes are shown above each node and bootstrap values from RAxML are shown below each node. Diamonds indicate support less than 80%. An “X” under a node indicates this node was not recovered in the RAxML maximum likelihood tree. Tree was rooted with the brown algae G. theta (Chlorophyta). The topology of the tree indicates that the last common ancestor of choanoflagellates and metazoans had a single tektin gene. Subsequent gene duplications gave rise to two and four tektin genes in the metazoan and bilaterian ancestor, respectively. For further information consult the legends for Figs. 3 and 4. Species abbreviations and accession numbers for each sequence are provided in Additional file 5. (PDF 530 kb) [file 12862_2019_1360_MOESM6_ESM.pdf]

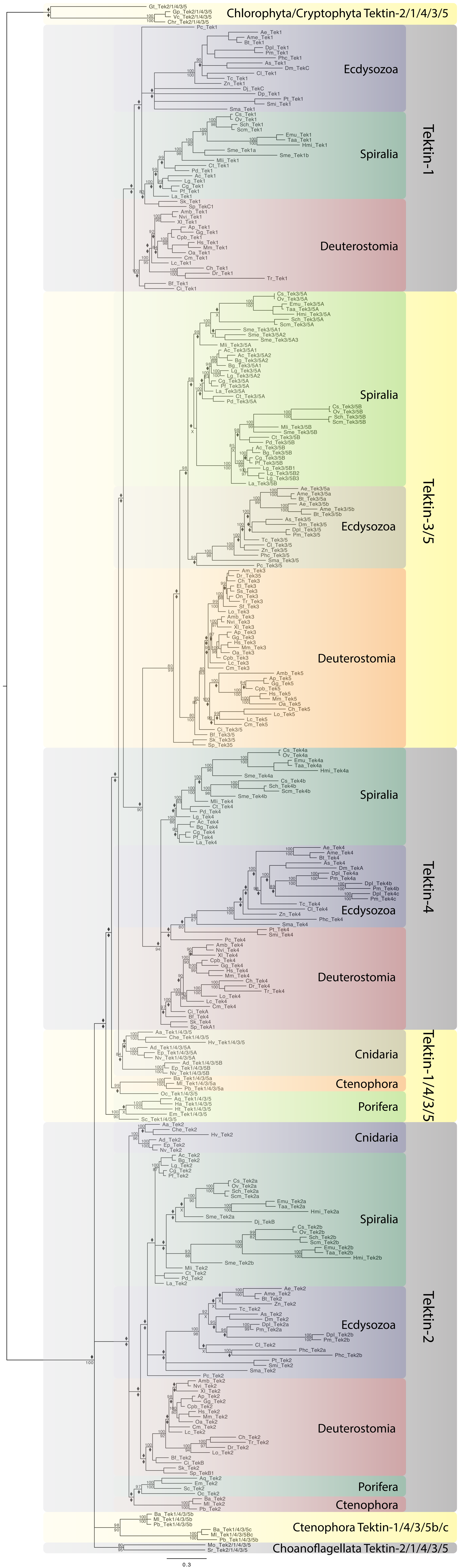

Chlorophyta/Cryptophyta Tektin-2/1/4/3/5
Ecdysozoa
Spiralia
Deuterostomia
Tektin-1
Spiralia
Ecdysozoa
Deuterostomia
Tektin-3/5
Spiralia
Ecdysozoa
Deuterostomia
Tektin-4
Cnidaria
Ctenophora
Porifera
Tektin-1/4/3/5
Cnidaria
Spiralia
Ecdysozoa
Deuterostomia
Porifera
Ctenophora
Tektin-2
Ctenophora Tektin-1/4/3/5b/c
Choanoflagellata Tektin-2/1/4/3/5
0.3
