# Supplementary figures and images for: Taxon-specific expansion and loss of tektins inform metazoan ciliary diversity
Source: BMC Evol Biol. 2019 Jan 31;19:40. doi: 10.1186/s12862-019-1360-0 (PMC6357514; doi:10.1186/s12862-019-1360-0)

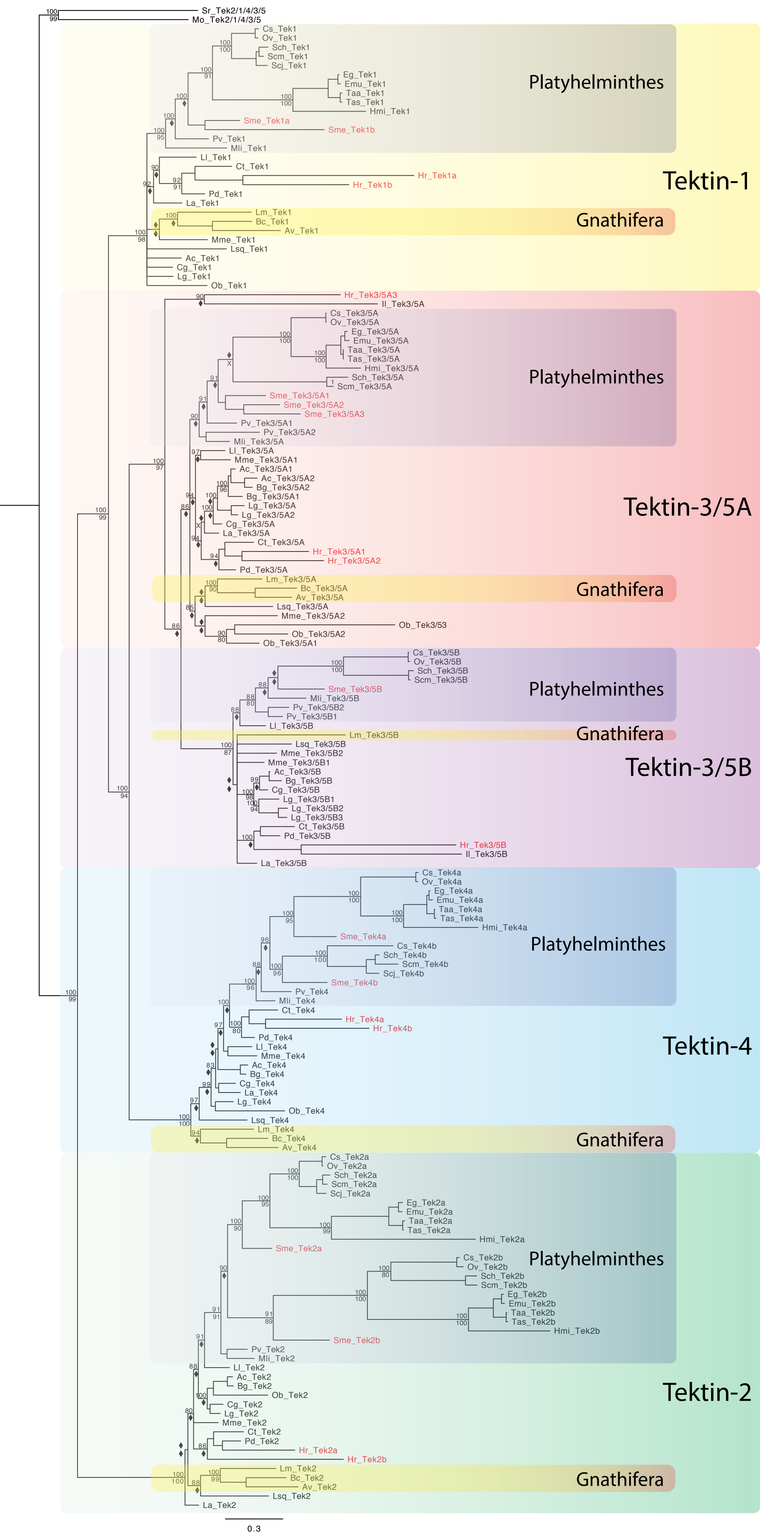

Supplement: Supplementary file 7 — Phylogenetic tree illustrating the tektin gene diversity in spiralians. This phylogenetic analysis is focused on spiralian tektins including the full complement of the greatly expanded and divergent tektin gene complements of Platyhelminthes and the leech H. robusta. Both Bayesian and Maximum Likelihood analyses were performed using Mr. Bayes and RAxML, respectively. Bayesian tree is shown. Node support is shown for non-terminal nodes. Posterior probability values from Mr. Bayes are shown above each node and bootstrap values from RAxML are shown below each node. Diamonds indicate support less than 80%. An “X” under a node indicates this node was not recovered in the RAxML maximum likelihood tree. Tree was rooted with choanoflagellate Tektins. The planarian S. mediterranea (Sme) and the leech H. robusta (Hr) independently evolved an identical expanded tektin gene complement of ten tektin genes from the five ancestral spiralian tektin genes. Some Tektins were subsequently lost in the parasitic Platyhelminthes. Platyhelminthes species are highlighted with boxes. S. mediterranea and H. robusta are highlighted with red text. Species abbreviations and accession numbers for each sequence are provided in Additional file 5. (PDF 689 kb) [file 12862_2019_1360_MOESM7_ESM.pdf]

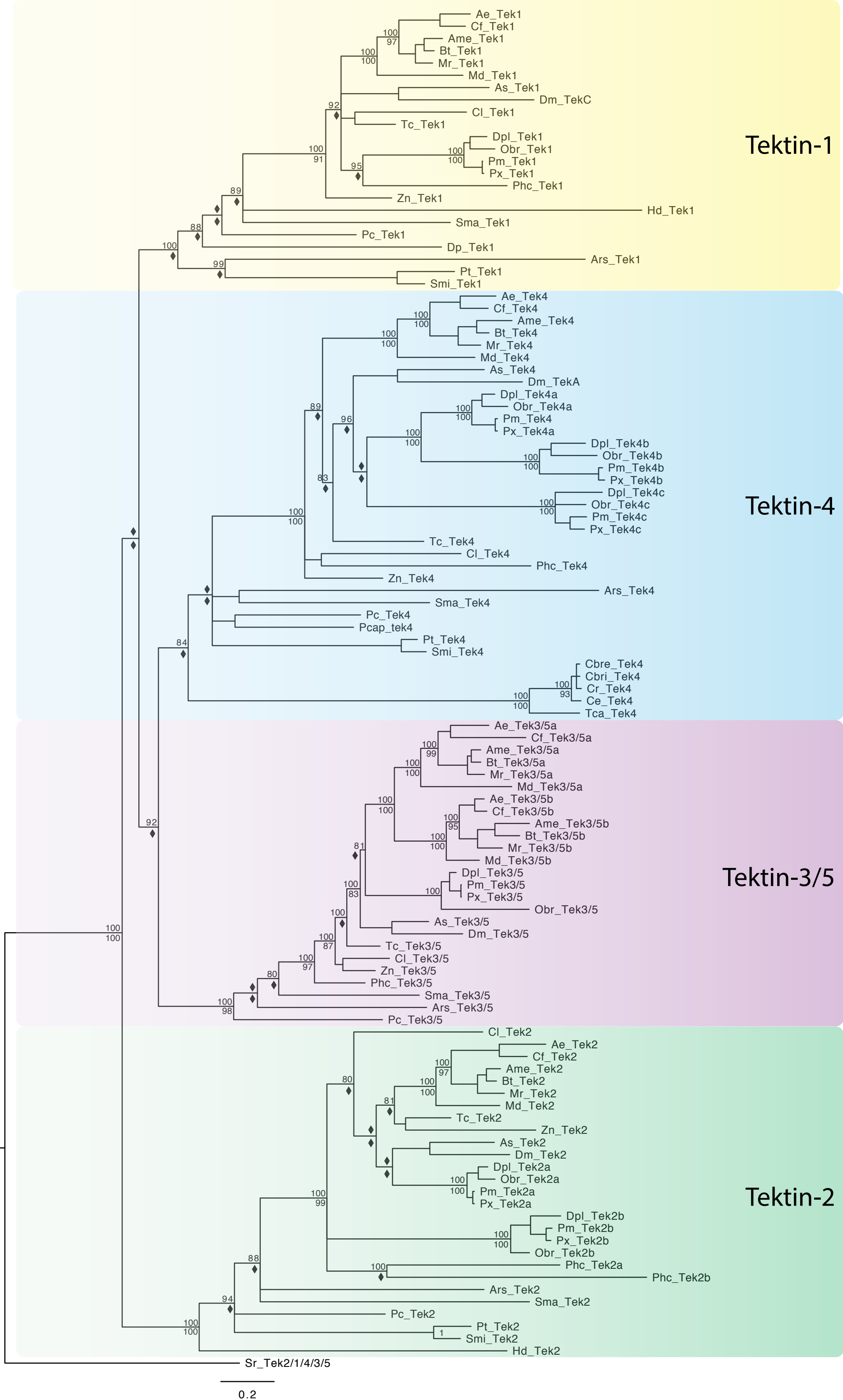

Supplement: Supplementary file 8 — Phylogenetic tree illustrating the tektin gene diversity in ecdysozoans. This phylogenetic analysis is focused on ecdysozoan tektins. Both Bayesian and Maximum Likelihood analyses were performed using Mr. Bayes and RAxML, respectively. Bayesian tree is shown. Node support is shown for non-terminal nodes. Posterior probability values from Mr. Bayes are shown above each node and bootstrap values from RAxML are shown below each node. Diamonds indicate support less than 80%. An “X” under a node indicates this node was not recovered in the RAxML maximum likelihood tree. Tree was rooted with choanoflagellate Tektin. Species abbreviations and accession numbers for each sequence are provided in Additional file 5. (PDF 659 kb) [file 12862_2019_1360_MOESM8_ESM.pdf]
