# Supplementary material for: Taxon-specific expansion and loss of tektins inform metazoan ciliary diversity
Source: BMC Evol Biol. 2019 Jan 31;19:40. doi: 10.1186/s12862-019-1360-0 (PMC6357514; doi:10.1186/s12862-019-1360-0)
Supplement: Supplementary file 9 — Comparison of this study vs. Nevers et al. [50] and Amos [20]. Comparison of findings of this study to phylogenetic analysis data by Nevers et al. [50] and Amos [20]. Species examined in both this study and at least one of the previous two are shown with the number of each Tektin class, and total number of Tektins found in each study. As the Nevers et al. [50] study used presence/absence data, a ‘+’ indicates presence while ‘0’ represents absence. Totals for Nevers et al. [50] are based on assumption that a ‘+’ is equal to one homolog. * indicates that different species from the same genus are compared. ** indicates different species from the same order or family are compared. *** indicates at least one Tektin did not group with any of the five recognized Tektins. † indicates that Nevers et al. [50] identified additional Tektin homologs that our study was unable to find. †† indicates the special case of human Tektins reported in Amos [20] (see Discussion). Green background indicates the previous study found the same number of Tektins as our study but with one or more misclassified. Red background indicates that the previous study found a different number of Tektins than our study. (PDF 660 kb) [file 12862_2019_1360_MOESM9_ESM.pdf]

This Study

<table>
<tr><th></th><th>Species</th><th>Tek-2</th><th>Tek-1</th><th>Tek-4</th><th>Tek-3</th><th>Tek-5</th><th>Total</th></tr>
<tr><td rowspan="6">Unicellular</td><td>Chlamydomonas reinhardtii</td><td colspan="5">1</td><td>1</td></tr>
<tr><td>Volvox carteri</td><td colspan="5">1</td><td>1</td></tr>
<tr><td>Trypanosoma brucei</td><td colspan="5">0</td><td>0</td></tr>
<tr><td>Tetrahymena thermophila</td><td colspan="5">0</td><td>0</td></tr>
<tr><td>Paramecium tetraurelia</td><td colspan="5">0</td><td>0</td></tr>
<tr><td>Salpingoeca rosetta</td><td colspan="5">1</td><td>1</td></tr>
<tr><td rowspan="4">Nonbilaterians</td><td>Trichoplax adhaerens</td><td>0</td><td colspan="4">0</td><td>0</td></tr>
<tr><td>Amphimedon queenslandica</td><td>1</td><td colspan="4">1</td><td>2</td></tr>
<tr><td>Nematostella vectensis</td><td>1</td><td colspan="4">2</td><td>3</td></tr>
<tr><td>Hydra vulgaris</td><td>1</td><td colspan="4">1</td><td>2</td></tr>
<tr><td rowspan="10">Deuterostomes</td><td>Strongylocentrotus purpuratus</td><td>1</td><td>1</td><td>1</td><td colspan="2">1</td><td>4</td></tr>
<tr><td>Branchiostoma floridae</td><td>1</td><td>1</td><td>1</td><td colspan="2">1</td><td>4</td></tr>
<tr><td>Ciona intestinalis</td><td>1</td><td>1</td><td>1</td><td colspan="2">1</td><td>4</td></tr>
<tr><td>Danio rerio</td><td>1</td><td>1</td><td>1</td><td>1</td><td>0</td><td>4</td></tr>
<tr><td>Takifugu rubripes/Tetraodon nigroviridis**</td><td>1</td><td>1</td><td>1</td><td>1</td><td>0</td><td>4</td></tr>
<tr><td>Xenopus (laevis/tropicalis)*</td><td>1</td><td>1</td><td>1</td><td>1</td><td>0</td><td>4</td></tr>
<tr><td>Gallus gallus</td><td>1</td><td>1</td><td>1</td><td>1</td><td>1</td><td>5</td></tr>
<tr><td>Ornithorhynchus anatinus</td><td>1</td><td>1</td><td>1</td><td>1</td><td>1</td><td>5</td></tr>
<tr><td>Homo sapiens</td><td>1</td><td>1</td><td>1</td><td>1</td><td>1</td><td>5</td></tr>
<tr><td>Mus musculus</td><td>1</td><td>1</td><td>1</td><td>1</td><td>1</td><td>5</td></tr>
<tr><td rowspan="2">Spiralians</td><td>Schistosoma mansoni</td><td>2</td><td>1</td><td>1</td><td colspan="2">2</td><td>6</td></tr>
<tr><td>Schistosoma japonicum</td><td>2</td><td>1</td><td>1</td><td colspan="2">2</td><td>6</td></tr>
<tr><td rowspan="10">Ecdysozoans</td><td>Caenorhabditis elegans</td><td>0</td><td>0</td><td>1</td><td colspan="2">0</td><td>1</td></tr>
<tr><td>Caenorhabditis briggsae</td><td>0</td><td>0</td><td>1</td><td colspan="2">0</td><td>1</td></tr>
<tr><td>Ixodes scapularis</td><td>0</td><td>0</td><td>0</td><td colspan="2">0</td><td>0</td></tr>
<tr><td>Daphnia pulex</td><td>0</td><td>1</td><td>0</td><td colspan="2">0</td><td>1</td></tr>
<tr><td>Pediculus humanus corporis</td><td>2</td><td>1</td><td>1</td><td colspan="2">1</td><td>5</td></tr>
<tr><td>Tribolium castaneum</td><td>1</td><td>1</td><td>1</td><td colspan="2">1</td><td>4</td></tr>
<tr><td>Apis mellifera</td><td>1</td><td>1</td><td>1</td><td colspan="2">2</td><td>5</td></tr>
<tr><td>Drosophila melanogaster</td><td>1</td><td>1</td><td>1</td><td colspan="2">1</td><td>4</td></tr>
<tr><td>Anopheles (sinensis/gambiae)*</td><td>1</td><td>1</td><td>1</td><td colspan="2">1</td><td>4</td></tr>
<tr><td>Papilio machaon/Bombyx mori**</td><td>2</td><td>1</td><td>3</td><td colspan="2">1</td><td>7</td></tr>
</table>

Nevers et al 2017

Species	Tek-2	Tek-1	Tek-4	Tek-3	Tek-5	Total
Chlamydomonas reinhardtii	0	0	0	+	0	1
Volvox carteri	0	0	0	0	+	1
Trypanosoma brucei	0	0	0	0	0	0
Tetrahymena thermophila	0	0	0	0	0	0
Paramecium tetraurelia	0	0	0	0	0	0
Salpingoeca rosetta	+	+	+	+	+	5†
Trichoplax adhaerens	0	0	0	0	0	0
Amphimedon queenslandica	+	+	0	0	0	2
Nematostella vectensis	+	+	0	0	0	2
Hydra vulgaris	+	+	+	+	0	4†
Strongylocentrotus purpuratus	0	+	+	+	0	3
Branchiostoma floridae	+	+	+	+	0	4
Ciona intestinalis	+	+	+	+	0	4
Danio rerio	+	+	+	+	0	4
*Takifugu rubripes/Tetraodon nigroviridis***	0	0	+	+	0	2
*Xenopus (laevis/tropicalis)**	+	+	+	+	0	4
Gallus gallus	+	+	+	+	+	5
Ornithorhynchus anatinus	+	+	0	+	+	4
Homo sapiens	+	+	+	+	+	5
Mus musculus	+	+	+	+	+	5
Schistosoma mansoni	+	+	0	+	+	4
Schistosoma japonicum	0	+	+	+	+	4
Caenorhabditis elegans	0	+	0	0	0	1
Caenorhabditis briggsae	0	+	0	0	0	1
Ixodes scapularis	0	0	0	0	0	0
Daphnia pulex	+	+	+	+	+	5†
Pediculus humanus corporis	+	0	0	+	+	3
Tribolium castaneum	+	0	0	+	+	3
Apis mellifera	+	+	0	+	+	4
Drosophila melanogaster	+	0	0	+	+	3
*Anopheles (sinensis/gambiae)**	+	0	0	+	+	3
*Papilio machaon/Bombyx mori***	+	+	+	+	+	5

Amos 2008

<table>
<tr><th>Species</th><th>Tek-2</th><th>Tek-1</th><th>Tek-4</th><th>Tek-3</th><th>Tek-5</th><th>Total</th></tr>
<tr><td>Chlamydomonas reinhardtii</td><td colspan="5">1</td><td>1</td></tr>
<tr><td>Volvox carteri</td><td colspan="5">NA</td><td>NA</td></tr>
<tr><td>Trypanosoma brucei</td><td colspan="5">NA</td><td>NA</td></tr>
<tr><td>Tetrahymena thermophila</td><td colspan="5">NA</td><td>NA</td></tr>
<tr><td>Paramecium tetraurelia</td><td colspan="5">NA</td><td>NA</td></tr>
<tr><td>Salpingoeca rosetta</td><td colspan="5">NA</td><td>NA</td></tr>
<tr><td>Trichoplax adhaerens</td><td colspan="5">NA</td><td>NA</td></tr>
<tr><td>Amphimedon queenslandica</td><td colspan="5">NA</td><td>NA</td></tr>
<tr><td>Nematostella vectensis</td><td colspan="5">NA</td><td>NA</td></tr>
<tr><td>Hydra vulgaris</td><td colspan="5">NA</td><td>NA</td></tr>
<tr><td>Strongylocentrotus purpuratus</td><td>1</td><td>1</td><td>1</td><td colspan="2">0</td><td>3</td></tr>
<tr><td>Branchiostoma floridae</td><td colspan="5">NA</td><td>NA</td></tr>
<tr><td>Ciona intestinalis</td><td>1</td><td>0</td><td>1</td><td colspan="2">0</td><td>2</td></tr>
<tr><td>Danio rerio</td><td>1</td><td>1</td><td>1</td><td>0</td><td>0</td><td>3</td></tr>
<tr><td>Takifugu rubripes/Tetraodon nigroviridis**</td><td>1</td><td>0</td><td>1</td><td>1</td><td>0</td><td>3</td></tr>
<tr><td>Xenopus (laevis/tropicalis)*</td><td>1</td><td>1</td><td>1</td><td>1</td><td>0</td><td>4</td></tr>
<tr><td>Gallus gallus</td><td colspan="5">NA</td><td>NA</td></tr>
<tr><td>Ornithorhynchus anatinus</td><td colspan="5">NA</td><td>NA</td></tr>
<tr><td>Homo sapiens</td><td>1</td><td>1</td><td>1</td><td>1</td><td>1</td><td>10††</td></tr>
<tr><td>Mus musculus</td><td>1</td><td>1</td><td>1</td><td>1</td><td>1</td><td>5</td></tr>
<tr><td>Schistosoma mansoni</td><td colspan="5">NA</td><td>NA</td></tr>
<tr><td>Schistosoma japonicum</td><td>1</td><td>1</td><td>1</td><td colspan="2">2</td><td>6***</td></tr>
<tr><td>Caenorhabditis elegans</td><td>0</td><td>0</td><td>0</td><td colspan="2">0</td><td>1***</td></tr>
<tr><td>Caenorhabditis briggsae</td><td>0</td><td>0</td><td>0</td><td colspan="2">0</td><td>1***</td></tr>
<tr><td>Ixodes scapularis</td><td colspan="5">NA</td><td>NA</td></tr>
<tr><td>Daphnia pulex</td><td colspan="5">NA</td><td>NA</td></tr>
<tr><td>Pediculus humanus corporis</td><td colspan="5">NA</td><td>NA</td></tr>
<tr><td>Tribolium castaneum</td><td colspan="5">NA</td><td>NA</td></tr>
<tr><td>Apis mellifera</td><td colspan="5">NA</td><td>NA</td></tr>
<tr><td>Drosophila melanogaster</td><td>1</td><td>0</td><td>0</td><td colspan="2">1</td><td>3***</td></tr>
<tr><td>Anopheles (sinensis/gambiae)*</td><td>1</td><td>0</td><td>0</td><td colspan="2">0</td><td>2***</td></tr>
<tr><td>Papilio machaon/Bombyx mori**</td><td colspan="5">NA</td><td>NA</td></tr>
</table>
